# Kondo-like phonon scattering in thermoelectric clathrates

**DOI:** 10.1038/s41467-019-08685-1

**Published:** 2019-02-21

**Authors:** M. S. Ikeda, H. Euchner, X. Yan, P. Tomeš, A. Prokofiev, L. Prochaska, G. Lientschnig, R. Svagera, S. Hartmann, E. Gati, M. Lang, S. Paschen

**Affiliations:** 1 Institute of Solid State Physics, Vienna University of Technology, Wiedner Hauptstr. 8-10, 1040 Vienna, Austria; 2 Helmholtz Institute Ulm for Electrochemical Energy Storage, Helmholtzstr. 11, 89081 Ulm, Germany; 3 Center for Micro- and Nanostructures, Nanocenter Campus Gußhaus, Vienna University of Technology, Gußhausstr. 25-25a, Gebäude CH, 1040 Vienna, Austria; 4 Physikalisches Institut, Goethe-Universität Frankfurt, Max-von-Laue-Straße 1, 60438 Frankfurt am Main, Germany

## Abstract

Crystalline solids are generally known as excellent heat conductors, amorphous materials or glasses as thermal insulators. It has thus come as a surprise that certain crystal structures defy this paradigm. A prominent example are type-I clathrates and other materials with guest-host structures. They sustain low-energy Einstein-like modes in their phonon spectra, but are also prone to various types of disorder and phonon-electron scattering and thus the mechanism responsible for their ultralow thermal conductivities has remained elusive. Our thermodynamic and transport measurements on various clathrate single crystal series and their comparison with ab initio simulations reveal an all phononic Kondo effect as origin. This insight devises design strategies to further suppress the thermal conductivity of clathrates and other related materials classes, with relevance for thermoelectric waste heat recovery and, more generally, phononic applications. It may also trigger theoretical work on strong correlation effects in phonon systems.

## Abstract (web summary)

Thermoelectric clathrates host guest atoms that can rattle inside their surrounding cages, yielding unusual phononic properties. Ikeda et al. show that ab initio calculations fail to account for thermodynamic and thermal transport data and propose a Kondo-like mechanism to explain the discrepancy.

## Introduction

**Fig. 1:**
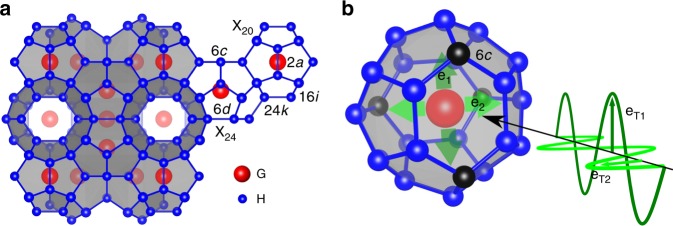
Clathrate crystal structure. **a** Type-I clathrate crystal structure G_8_H_46_. Per unit cell 8 guest atoms (G, red) are situated in two different cages (smaller dodecahedra X_20_ and larger tetrakaidecahedra X_24_) built by the host (H, blue) atoms. The different crystallographic sites are labeled. **b** Sketch of an X_24_ cage with the two soft rattling directions *e*_1_ and *e*_2_ within the easy plane parallel to the two hexagonal faces of the cage. Each soft direction is parallel to the longest secant of one of the two hexagons, both defined by the 6*c* site (black atoms) of the structure. Incoming acoustic phonons with the two different transverse polarizations *e*_T1_ and *e*_T2_ are sketched by the sinusoidal waves

The increasing world wide energy consumption and the associated climate change call for enhancing the overall energy efficiency of technological energy conversion processes. Thermoelectrics^1–6^ can convert waste heat into electricity and could thus contribute to such an efficiency increase. The perfect thermoelectric material combines low phonon thermal conductivity with high electrical conductivity and high thermopower. Materials classes with cage-like structures such as the clathrates^7,8^ or skutterudites^4,9^ show surprisingly low phonon thermal conductivities *κ*_ph_, even in perfect single crystals. Type-I clathrates G_8_H_46_, that show promising thermoelectric figures of merit^5,10,11^, consist of host atoms (H) that encapsulate guest atoms (G) in a framework of oversized cages (Fig. 1a). Recent inelastic neutron and X-ray scattering studies uncovered energetically low-lying optical phonon modes which were attributed to a so-called rattling motion of the guest atoms in the cages and are thus referred to as Einstein modes^12–14^. Similar observations were also made for skutterudites^9^ and clathrate hydrates^15^. A severe flattening of the acoustic phonon branches at energies near the optical modes was observed^12–14^, and attributed to a finite coupling between the guest atoms and the host cages. Early thermal conductivity studies on type-I clathrates^16,17^, inspired by investigations on glasses^18^, tried to capture this guest-host coupling by including terms for resonance scattering – originally proposed to describe the resonance interaction between phonons and non-paramagnetic defects^19^, and for scattering from tunneling states^18^ – into phenomenological treatments^20^. Whereas empirical multi-parameter fits including these terms can indeed model thermal conductivity data of several type-I clathrates below about 50 K (refs. ^16,17^), inconsistencies with other results have been pointed out more recently. Notably, such modeling can neither explain the exceptionally long phonon lifetimes^12,21^ nor the large thermal conductivity differences between structurally very similar n- and p-type versions of these materials^22^. The recent achievement of ab initio calculations of the thermal conductivity of a type-I clathrate, based on intrinsic phonon-phonon Umklapp scattering processes^23^, represents a major step forward. It showed that, unlike in the resonance scattering picture, the phonon lifetimes are reduced in a wide frequency range. Nevertheless, also DFT calculations can explain experimental thermal conductivity data only at low temperatures, but strongly undershoot them at high temperatures.

Here we propose an alternative mechanism, a Kondo-like phonon-phonon interaction, that can explain the difference as the disentanglement of Kondo-coupled acoustic and rattling phonon modes above the phonon Kondo temperature. In general terms, the Kondo effect describes non-commutative scattering of an extended wave from a localized entity with internal degree of freedom. It has been studied in many different settings, including magnetic, orbital, charge, and local vibrational degrees of freedom (see Supplementary Note 8 for more details). However, to the best of our knowledge, it has never been studied in an all phononic context.

## Results

### Steps beyond the state-of-the-art

**Fig. 2:**
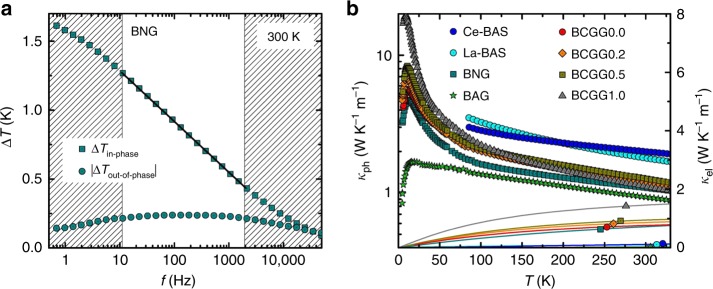
Thermal conductivity data of type-I clathrate single crystals. **a** Exemplary frequency scan in the 3*ω* setup. Rectangles show the in-phase, circles the out-of-phase contribution of the temperature oscillation Δ*T*. The expected linear-in-logarithmic-frequency dependence of the in-phase temperature oscillation of the heater/thermometer line, that is observed at intermediate frequencies (between the hatched areas), is measured with an extremely low noise level. The slope of this dependence (full line) is inversely proportional to the thermal conductivity *κ* of the studied material. **b** Temperature-dependent thermal conductivity of all studied materials. The data points represent the phonon thermal conductivities *κ*_ph_, calculated by subtracting an electronic contribution *κ*_el_ (lines and identifier symbols, right axis) from the total measured *κ*. Each data point above 80 K is determined from the slope of an isothermal Δ*T* vs log *f* curve (straight line in **a**). All sample compositions are given in the Supplementary Table 1

To demonstrate the all phononic Kondo effect, we had to go beyond the state-of-the-art in thermoelectric clathrates’ research in several respects. Firstly, to overcome the bottleneck of large uncertainties and systematic errors of standard thermal conductivity measurements at elevated temperatures, we developed a high-precision implementation (Fig. 2a and “Methods” section) of the 3*ω* technique^24^. A second key ingredient for our work are sample series of single crystals, which allow us to pin down the dominating instrinsic phonon scattering mechanisms by employing an empirical model^20^, modified to be in line with the most recent findings^21,23^ on the phonon band structure and phonon dynamics in type-I clathrates. Thirdly, we compare both the thermal conductivity data and thermodynamic data to ab initio lattice dynamics calculations, thereby providing compelling evidence for the phonon Kondo effect.

### Phonon thermal conductivities

We start by giving an overview of the phonon thermal conductivities *κ*_ph_ of all type-I clathrate single crystals studied here (Fig. 2b). Although type-I clathrates are often referred to as model systems for the enigmatic phonon-glass electron-crystal concept^1^, the most prominent feature of *κ*_ph_(*T*) is a large crystal-like maximum. It is narrow and occurs at a surprisingly low temperature of only about 10 K. As will be further detailed below, this narrow low-temperature maximum is a strong indication for the phonon transport not being controlled by the natural energy scale *k*_B_Θ_D_ of the Debye temperature Θ_D_ but by a much smaller energy scale *k*_B_Θ_E_.

**Fig. 3:**
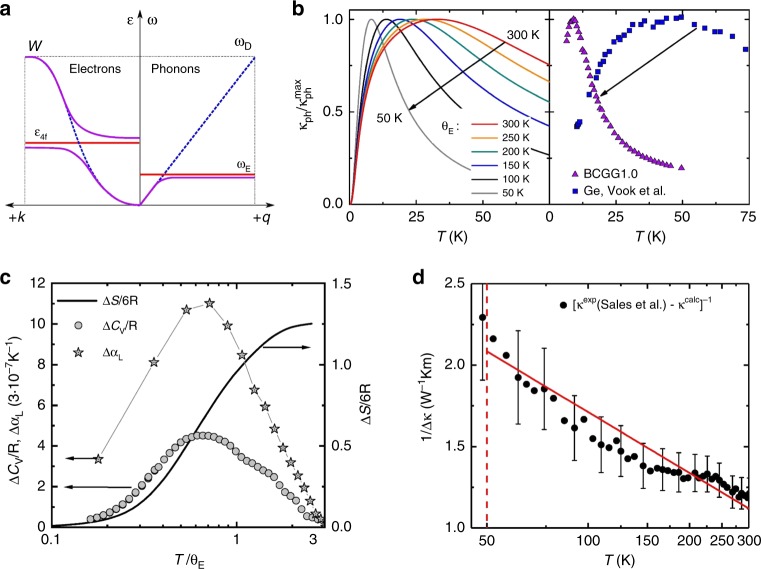
Comparison of spin and phonon Kondo effect. **a** Schematic dispersion relations for electrons in heavy electron systems (left) and phonons in an analogously defined “heavy” phonon system (right). The blue and red curves represent the non-interacting dispersive and localized entities, respectively, the violet curves the hybridized interacting states. As function of temperature, the system evolves from non-interacting well above the Kondo temperature to interacting well below it. For simplicity, phonon branches above *ω*_E_ are neglected. This is justified in real clathrates by the presence of multiple Einstein modes, resulting in multiple anticrossings^13, 14^. The Debye model (blue line, right) assumes *ω* = *v*_s_*q* where *v*_s_ is the sound velocity. The new dispersion relation (violet, right) is characterized by the group velocity *v*_g_ = ∂*ω*/∂*q*. It equals the sound velocity only at low wave vectors and frequencies. **b** Temperature-dependent phonon thermal conductivity, normalized to its maximum, calculated using a modified Callaway model (Supplementary Note 1) for various Einstein temperatures Θ_E_ (left) and corresponding data for the Ge-based type-I clathrate BCGG1.0 (Supplementary Table 1) and elemental Ge, electron irradiated and annealed at 77 K to similar defect densities^64^ (right). **c** Specific heat and thermal expansion phonon Kondo anomaly, obtained by subtracting the experimental from the theoretical specific heat and thermal expansion curves of Ba_8_Ga_16_Ge_30_ (left axis, see Supplementary Figs. 1b and 2b and Methods) and the corresponding entropy (right axis). **d** The inverse difference of calculated and experimental^41^ phonon thermal conductivities of Ba_8_Ga_16_Ge_30_ (Supplementary Fig. 3b), showing the −ln *T* hallmark (full red line) of incoherent Kondo scattering in the spin Kondo effect above the Kondo temperature (dashed vertical line). The error bars represent the error of ±3% specified for the thermal conductivity data^41^

To visualize the effect of a reduced Debye temperature, we use a simple phenomenological lattice thermal conductivity model: A modified version of the standard Callaway model^20^, where we replaced the Debye temperature Θ_D_ by a variable temperature Θ_E_ (Supplementary Note 1). The left panel of Fig. 3b shows temperature dependences of normalized phonon thermal conductivities calculated within this model for a series of different characteristic energies *k*_B_Θ_E_, keeping fixed scattering rates $\tau _{\mathrm{D}}^{ - 1}$ for defect scattering, $\tau _{\mathrm{B}}^{ - 1}$ for boundary scattering, and $\tau _{{\mathrm{ph}} - {\mathrm{el}}}^{ - 1}$ for phonon-electron scattering. Resonance scattering is of minor importance here (Supplementary Fig. 4). With decreasing Θ_E_ the broad maximum sharpens and is shifted to lower temperatures. This is attributed to a strongly enhanced Umklapp scattering rate $\tau _{\mathrm{U}}^{ - 1}$ (Supplementary Note 1) that results from successively limiting the energy range of the contributing acoustic phonons as Θ_E_ decreases.

This effect can be directly observed by experiments. The right panel of Fig. 3b compares the phonon thermal conductivities of a Ge-based type-I clathrate single crystal (BCGG1.0, see Supplementary Table 1) and single crystalline Ge, irradiated^25^ to a similar defect concentration. It is clear that the clathrate is governed by a strongly reduced energy scale. Both materials have similar Debye temperatures (Supplementary Note 2), but the clathrate has, in addition, a low-lying Einstein temperature Θ_E_, that apparently controls the behavior.

### Dominating role of Umklapp scattering

**Fig. 4:**
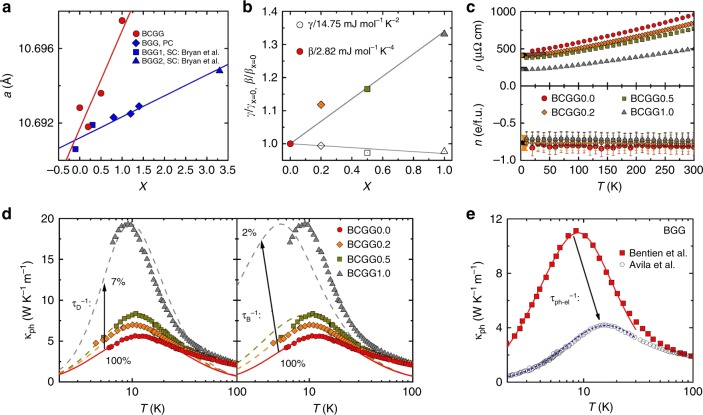
Scattering contributions in clathrate single crystal series. **a** Lattice parameter vs *x* for Ba_8_Cu_4.8_Ge_41.2−*x*−*y*_□_*y*_Ga_*x*_ (BCGG*x*, Supplementary Table 1) and comparison with the series Ba_8_Ga_14+*x*_Ge_32−*x*_ (BGG are polycrystals synthesized by us, BGG1 are single crystals 4 and 6 from ref. ^65^, and BGG2 is a single crystal from ref. ^66^), which was shifted downwards by 89.6 mÅ for better readability. For the linear fits see Supplementary Note 3. **b** Coefficients *γ* and *β* of linear fits *C*_p_/*T* = *γ* + *βT*^2^ to the specific heat data, normalized to their respective value for *x* = 0, of Ba_8_Cu_4.8_Ge_41.2−x−y_□_*y*_Ga_*x*_ below 3.5 K (not shown) vs *x*. **c** Electrical resistivity (top) and charge carrier concentration (bottom), determined from the Hall coefficient in a one-band model, *R*_H_ = 1/(*ne*), as function of temperature for all Ba_8_Cu_4.8_Ge_41.2−*x*−*y*_□_*y*_Ga_*x*_ samples. **d** Phonon thermal conductivity vs temperature for all Ba_8_Cu_4.8_Ge_41.2−*x*−*y*_□_*y*_Ga_*x*_ samples (symbols), together with fits with the modified Callaway model (Supplementary Note 1) to the *x* = 0 data (red full line) and simulations (dashed lines) using the parameters of the *x* = 0 fit except for the defect scattering rate $\tau _{\mathrm{D}}^{ - 1}$ (left) and the boundary scattering rate $\tau _{\mathrm{B}}^{ - 1}$ (right). The respective scattering rate was decreased to the indicated percentage in direction of the arrow. The speed of sound of each sample was determined from *β*, the Einstein temperature Θ_E_ from bell-shaped contributions to *C*_p_/*T*^3^ versus log*T*. **e** Phonon thermal conductivity vs temperature for two different Ba_8_Ga_16−*x*_Ge_30+*x*_ samples from the literature^30, 31^, with lines corresponding to our fit to the data from ref. ^30^ and a simulation using essentially the same parameters except for a strongly enhanced phonon-electron scattering rate $\tau _{{\mathrm{ph}} - {\mathrm{el}}}^{ - 1}$ for the data from ref. ^31^

Next, we prove, with two clathrate single crystal series, that the strong Umklapp scattering due to the new low-energy scale indeed dominates the lattice thermal conductivity in a wide temperature range. In the first such series, Ba_8_Cu_4.8_Ge_41.2−*x*−*y*_□_*y*_Ga_*x*_ (*x* = 0, 0.2, 0.5, 1.0, *y* = 1.2 − *x*, see BCGG*x* in Supplementary Table 1), an increasing Ga content *x* is accompanied by a decreasing content *y* of host atom vacancies □. This is supported by the increase of the lattice parameter *a* (Fig. 4a) and the Hall mobility *μ*_H_ = *R*_H_/*ρ* = 1/(*neρ*) (combination of the two panels in Fig. 4c) with *x*, as well as by the charge neutrality of this substitution (both the electronic *γ* term of the specific heat and the charge carrier concentration *n* are essentially independent of *x*, see Fig. 4b, c bottom), as discussed in the Supplementary Note 3. Because the mass difference between Ga and Ge is very small, Ga represents a much weaker scattering potential^26^ in Ba_8_Cu_4.8_Ge_41.2−*x*−*y*_□_*y*_Ga_*x*_ than a vacancy. Thus, we expect a decrease of defect scattering with *x*. The low-temperature phonon thermal conductivity *κ*_ph_(*T*) of all samples in this series shows a maximum at low temperatures that is systematically enhanced at nearly constant temperature with increasing *x* (Fig. 4d left). This trend is indeed well reproduced by a decrease of the point-defect scattering rate $\tau _{\mathrm{D}}^{ - 1}$ with *x*, as seen by the good agreement of simulation and data for this case (full and dashed lines in Fig. 4d left). A much less satisfying agreement is observed if the boundary scattering rate $\tau _{\mathrm{B}}^{ - 1}$ is allowed to change with *x* instead (Fig. 4d right). The phonon-electron scattering rate $\tau _{{\mathrm{ph}} - {\mathrm{el}}}^{ - 1}$ can be assumed to be *x* independent because of the above discussed charge neutrality. Above about 50 K, however, the *κ*_ph_(*T*) data for different *x* merge (Fig. 4d left), indicating that scattering from point defects has become negligible. This can be rationalized by comparing the (Debye) phonon wavelength *λ* = 2*πv*_s_ / *ω*, where *v*_s_ is the sound velocity (related to *β*, Fig. 4b), with the size of the scattering center. A vacancy with the distortion surrounding it was estimated to have a diameter *d* ≈ 5 Å (ref. ^27^). Strong point-defect scattering of Rayleigh type, with an *ω*^4^ dependence, occurs only if *λ* is at least an order of magnitude larger than *d*, corresponding to phonon energies *ħω* of 2.5 meV (30 K) and below. At much larger energies (and temperatures), defect scattering should be weak and frequency independent^28^.

The second sample series is the prototypical clathrate Ba_8_Ga_16−*x*_Ge_30+*x*_ that has been much investigated in the past. It is an ideal system to study the importance of phonon-electron scattering because it has a low and essentially constant amount of point defects (as seen from the above, shown in ref. ^26^, and explained and demonstrated in the Supplementary Note 9 and Supplementary Fig. 5, respectively, Ga does not act as strong point defect in Ge clathrates) but a charge carrier concentration that varies strongly with *x* (ref. ^29^).

Indeed, different Ba_8_Ga_16−*x*_Ge_30 + *x*_ single crystals reported in the literature^30,31^ show severely different *κ*_ph_(*T*) at low temperatures. Figure 4e replots two extreme cases. The red line is our low-temperature fit (*T* < Θ_E_/2) with the modified Callaway model discussed in Supplementary Note 1 to the data of ref. ^30^. It takes Umklapp scattering $\left( {\tau _{\mathrm{U}}^{ - 1}} \right)$, defect scattering $\left( {\tau _{\mathrm{D}}^{ - 1}} \right)$, boundary scattering $\left( {\tau _{\mathrm{B}}^{ - 1}} \right)$, and phonon-electron scattering $\left( {\tau _{{\mathrm{ph}} - {\mathrm{el}}}^{ - {\mathrm{1}}}} \right)$ into account. The latter is calculated from the reported materials properties^30^ as discussed in Supplementary Note 1. Interestingly, the *κ*_ph_(*T*) data of a different Ba_8_Ga_16−*x*_Ge_30+*x*_ single crystal^31^ can be very well reproduced by strongly increasing $\tau _{{\mathrm{ph}} - {\mathrm{el}}}^{ - 1}$ and by only slightly adjusting $\tau _{\mathrm{U}}^{ - 1}$ (dashed line in Fig. 4e). Above about 50 K, the differences in *κ*_ph_ caused by a different $\tau _{{\mathrm{ph}} - {\mathrm{el}}}^{ - 1}$ vanish. This observation is in line with the fact that phonon-electron scattering can only occur for phonons with a wave vector *q* smaller than twice the Fermi wave vector *k*_F_. For the crystal of ref. ^30^ we estimate this to hold below about 140 K (Supplementary Note 2). At higher temperatures phonon-electron scattering is unlikely to be relevant.

A suppression of large low-temperature differences in *κ*_ph_(*T*) at higher temperatures has also been seen in other type-I clathrate single crystal series^32,33^, but precise high-temperature data on these are not available to date.

Taking both our clathrate series together we have managed to rule out the influence of defect and phonon-electron scattering on *κ*_ph_(*T*) of various type-I clathrates above 50 K. In addition, boundary scattering cannot contribute significantly in single crystals at these temperatures. Thus, at elevated temperatures, *κ*_ph_ is dominated by intrinsic phonon-phonon (Umklapp) scattering. This allows us to pin down its microscopic origin, as shown in what follows.

### Universal scaling

**Fig. 5:**
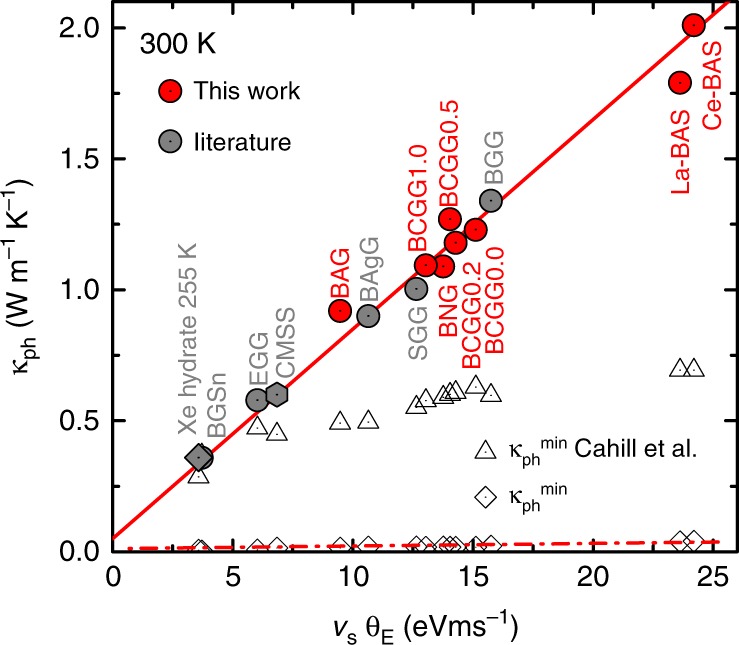
Universal scaling of the intrinsic phonon thermal conductivity of phonon Kondo compounds. Phonon thermal conductivities vs the product of sound velocity and lowest-lying Einstein temperature (Supplementary Note 5) for single crystalline intermetallic clathrates measured within this work (red circles), together with published data on intermetallic clathrates (grey circles), a Xe-filled ice hydrate (grey square), and the tetrahedrite Cu_10.6_Mn_1.4_Sb_4_S_13_ (CMSS, grey hexagon; see Supplementary Table 1 for all sample compositions and references). The open triangles are the minimum *κ*_ph_ values derived using *κ*_min_ of ref. ^45^. The open squares are the minimum *κ*_ph_ values estimated as discussed in Supplementary Note 1

Remarkably, a broad range of clathrates, including even a gas hydrate^15,34–36^, shows a universal scaling of the room-temperature phonon thermal conductivity with the product of sound velocity and Einstein temperature of the lowest-lying rattling mode(s), *κ*_ph_ ∝ *v*_s_Θ_E_ (Fig. 5). The simple kinetic gas relation $\kappa _{{\mathrm{ph}}} = c_{\mathrm{v}}v_{\mathrm{s}}^2\tau {\mathrm{/}}3$ predicts *κ*_ph_ to depend on the square of the sound velocity. In the Debye model the sound velocity is proportional to the Debye temperature and thus $\kappa _{{\mathrm{ph}}} \propto v_{\mathrm{s}}^2 \propto \Theta _{\mathrm{D}}^2$ is expected for simple Debye solids. The modified scaling $\kappa _{{\mathrm{ph}}} \propto v_{\mathrm{s}}{\mathrm{\Theta }}_{\mathrm{E}} \propto v_{\mathrm{s}}^2 \cdot \left( {{\mathrm{\Theta }}_{\mathrm{E}}{\mathrm{/\Theta }}_{\mathrm{D}}} \right)$ shows that the above discussed energy renormalization is universal in clathrates.

### Analogy with spin Kondo effect

A similar energy renormalization is seen in heavy fermion metals, where the (spin) Kondo effect rescales the Fermi temperature *T*_F_ to the (spin) Kondo temperature *T*_K_ (refs. ^37,38^). Figure 3a illustrates this analogy with schematic dispersion relations for electrons and phonons. In a band picture for heavy electron systems (left) a broad conduction band (blue) hybridizes with a flat, essentially non-dispersing 4*f* band of (renormalized) energy $\epsilon _{4f}$ (red). The hybridized bands (violet) are extremely flat near $\epsilon _{4f}$, corresponding to quasiparticles with strongly renormalized effective masses. In the phonon case the strongly dispersing acoustic phonon mode, approximated here by the linear dispersion of the Debye model, takes the role of the broad conduction band, and the flat Einstein-like rattling mode at *ħω*_E_ = *k*_B_Θ_E_ corresponds to the narrow 4*f* band. The resulting hybridized band is again extremely flat in large portions of the Brillouin zone, giving rise to “heavy” phonons with extremely low group velocity *v*_g_ at finite wave vectors. Even though the resulting *T* = 0 dispersion relation may look similar to the one obtained from ab initio lattice dynamics simulations^23^, there is an important difference: Being a strong correlation phenomenon, the phonon Kondo effect has a characteristic temperature dependence. Well above the Kondo temperature, the interacting states (violet) go over to the non-interacting ones (red and blue), an effect referred to as crossover from infrared slavery to asymptotic freedom^38^, which is absent in the lattice dynamics simulations (see also “Discussion” section).

The fact that the observed universal scaling of *κ*_ph_ still contains *v*_s_ (to linear power) is attributed to the absence of a Fermi level in phonon systems. Whereas the electrical resistivity in heavy fermion metals is dominated by the heavy electrons at the Fermi wavevector *k*_F_, phonons of all wave vectors, including long-wavelength ones propagating with *v*_s_, contribute to *κ*_ph_.

Finally, we compare specific heat, thermal expansion, and thermal conductivity data for the prototypical clathrate Ba_8_Ga_16_Ge_30_ with ab initio lattice dynamics calculations (see Methods) and reveal key characteristics of the Kondo effect. For the specific heat, the agreement between experiment and theory is excellent at low and high temperatures, but a distinct deviation is seen in between (Supplementary Fig. 1b). This difference gives rise to an anomaly near Θ_E_ (Fig. 3c). It releases an entropy of order *R* ln2 per rattling atom at the 6*d* site. This is the behavior expected for any Kondo effect involving a 2-fold degenerate localized entity: Its degeneracy is lifted by the Kondo interaction, giving rise to the above entropy release. The entropy reaches 0.65 ln2 at 40 K. This temperature is considered as good estimate of the Kondo temperature^39^, which is thus found to be comparable to Θ_E_. The experimental thermal expansion is sizeably smaller than the theoretical prediction below 150 K, but agrees well with it above this temperature (Supplementary Fig. 2b). The difference between the theoretical and experimental curve closely resembles the specific heat anomaly (Fig. 3c). These discrepancies in specific heat and thermal expansion translate into a corresponding discrepancy in the mode-averaged Grüneisen parameter: Ab initio calculations predict an upturn at low temperatures, an effect usually associated with enhanced phonon anharmonicity at low frequencies, that is however absent in the experimental curve (Supplementary Fig. 2c). Finally, the experimental phonon thermal conductivity is well described by the ab initio calculations below 50 K, but severely overshoots them at higher temperatures (Supplementary Fig. 3b). The difference, plotted as inverse to represent a thermal resistivity (Fig. 3d), increases with decreasing temperature, with a slow (about −ln *T*) dependence, the hallmark of incoherent Kondo scattering above *T*_K_ in the original spin Kondo effect^37,38^.

## Discussion

To understand these results, it is important to assess the limitations of the used ab initio calculations (see “Methods” section). The phonon density of states (DOS) and the specific heat are calculated in the harmonic approximation, the thermal expansion in the quasi-harmonic approximation, and the thermal conductivity from anharmonic interatomic force constants^23^. The latter thus takes phonon-phonon interactions to lowest order into account. However, temperature dependences resulting from strong phonon correlation effects, most notably the Kondo disentanglement of acoustic and rattling modes above the phonon Kondo temperature, cannot be captured by these simulations. This is why the comparison of experimental data and such calculations can be used to extract the characteristic temperature dependences due to the correlations (somewhat like non-*f* reference materials are used as background to reveal Kondo physics in heavy electron compounds – unfortunately, empty type-I clathrates without the rattling atoms, that would represent such background here, do not exist). Specifically, the temperature-dependent specific heat is overestimated by the calculations as, unlike in the Kondo effect, no entropy is released by Bose populating temperature-independent anticrossings (circles in Fig. 3c). The thermal expansion calculations do not contain the non-trivial temperature dependence of the anharmonicities due to the phonon Kondo interaction, which leaves a corresponding imprint in the thermal expansion difference (stars in Fig. 3c). Finally, the strong Umklapp scattering that is captured by the thermal conductivity calculations does not contain the weakening above *T*_K_ characteristic of an asymptotically free theory (Fig. 3d).

Further support, independent of any theoretical modeling, comes from recent inelastic neutron scattering experiments. They reveal unexpected changes of the optical dynamical structure factors as function of temperature^21^ that could be an indication of such a disentanglement of interacting acoustic and rattling modes at high temperatures. All these observations provide strong evidence for a previously unknown correlation effect – an all phononic Kondo effect – as microscopic origin of the peculiar thermodynamic and thermal transport behavior of clathrates.

We do not want to conclude without proposing a possible microscopic realization of the phonon Kondo effect in clathrates. The rattling motion of the guest atoms at the crystallographic 6*d* site within a soft plane^12,31,40^ in the tetracaidecahedra (Fig. 1b) is known to have the lowest frequency^23^. Within this plane, due to the four-fold symmetry of the potential well, rattling occurs preferentially along two perpendicular soft directions^22,41,42^, which we refer to as 1 and 2. The two corresponding rattling modes (*e*_1_ and *e*_2_ in Fig. 1b), which are degenerate in energy for symmetry reasons, are thus identified as the pseudospins of the Kondo model (they represent spin-up and spin-down states in the spin Kondo effect). They can be approximated as simple Einstein oscillators, with the first excited state corresponding to the Einstein temperature Θ_E_ observed in inelastic scattering experiments^12–14^. The two polarizations of the transverse acoustic phonons represent the corresponding degree of freedom of the itinerant species (*e*_T1_ and *e*_T2_ in Fig. 1b, thus representing the conduction electrons in the spin Kondo effect). The hybridization of the local (rattling) and extended (acoustic) phonon modes has been observed in inelastic neutron and X-ray scattering experiments^12–15,34^. A spin flip process in the spin Kondo effect corresponds to a scattering process with polarization change in the phonon Kondo effect. It can be visualized as follows: Assume the guest atom rattles in mode *e*_1_ and a propagating phonon of polarization *e*_T2_ “hops” onto the cage, creating a distortion of the cage that resembles the effect of mode *e*_2_. This additional cage distortion will facilitate a change of rattling direction from *e*_1_ to *e*_2_, and an accompanying change in polarization of the outgoing acoustic wave from *e*_T2_ to *e*_T1_. Such an interaction of two modes with “opposite” polarization is analogous to an antiferromagnetic exchange interaction in the spin Kondo effect. Further details as well as a suggestion for a mapping of these ingredients onto a Kondo-type Hamiltonian are given in the Supplementary Note 8. We hope that our discovery will be taken up by a broad community of theorists, to develop both ab initio and model-based approaches that can describe the unusual observed phonon properties.

Our discovery of Kondo physics in an all phononic system is not only of fundamental interest (see discussion of non-canonical Kondo physics in other settings in Supplementary Note 8), but also has practical implications. First, it gives a concise description of how to tailor rattling materials for thermoelectric applications at elevated temperatures, which are most relevant for waste heat-recovery applications: Materials with the lowest possible Einstein and thus phonon Kondo temperatures should be found. Microscopically, this might be achieved by changing the mass and size of the guest atoms, but also by structural disorder on the guest site^43,44^ and/or tailored guest-host charge transfer^43^.

An equally striking consequence is that Cahill’s definition of the minimum thermal conductivity $\kappa _{{\mathrm{ph}}}^{{\mathrm{min}}}$ (ref. ^45^), that is being referred to so extensively, needs to be reconsidered. With the energy renormalization also $\kappa _{{\mathrm{ph}}}^{{\mathrm{min}}}$ is drastically reduced (Supplementary Note 1). This implies that for the materials in Fig. 5 there is still room for further improvement as their phonon thermal conductivities all lie well above their respective new $\kappa _{{\mathrm{ph}}}^{{\mathrm{min}}}$ value (dashed red line in Fig. 5). Further lowering the *κ*_ph_ of a given material could, for instance, be realized by nanostructuring or the introduction of dense dislocation arrays^46^. Long-wavelength phonons that are only weakly affected by the phonon Kondo effect could, at high temperatures, be effectively scattered by nanostructures that are small compared to their mean free path. Indeed, a few clathrates and skutterudites appear to violate the original Cahill $\kappa _{{\mathrm{ph}}}^{{\mathrm{min}}}$ limit, though this has not been explicitly recognized in these works (Supplementary Note 2). We expect our results to trigger more systematic efforts along these lines.

The occurrence of low-lying Einstein-like phonon modes that interact with acoustic phonons is not limited to the class of clathrates and related cage-like materials. For the recently discovered family of Cu_12−*x*_M_*x*_Sb_4_S_13_ (M = transition metal) tetrahedrites, optical phonon branches involving out-of-plane vibrations of the three-fold coordinated Cu ions were predicted by ab initio calculations and were suggested as origin of the low thermal conductivities^47^. Interestingly, *κ*_ph_ of this material fits perfectly into our universal scaling plot (Fig. 5). Other candidate materials are, e.g., PbTe (ref. ^48^), Bi_2_Te_3_ (ref. ^49^), BiCu(Se,Te)O (ref. ^50^), Cu_3_SbSe_3_ (ref. ^51^), and rattling-induced superconductors such as the *β*-pyrochlore oxides^52^, dodecaborides^53^, or VAl_10_ (ref. ^54^), and possibly even amorphous and glassy materials^55^. Detailed investigations, such as presented here, are needed to test whether also in these materials the phonon Kondo effect is at work.

Phonon Kondo systems transfer heat largely via low-frequency phonons of long mean free paths. As such they may be seen as promising intrinsic thermocrystals, for applications such as heat waveguides or thermal diodes in the emerging field of phononics^56^.

## Methods

### Synthesis and structural characterization

As starting material for the single crystal growth of BCGG*x*, two cylindrical rods with the same nominal composition Ba_8_Cu_4.8_Ge_41.2−*x*−*y*_□_*y*_Ga_*x*_ (BCGG*x*) were prepared for each sample with *x* = 0.0, 0.2, 0.5, and 1.0 from high-purity elements using a high-frequency induction furnace. One rod with 7 mm in diameter and 60 mm in length served as the feed rod, the other one with the same diameter and 20 mm in length as the seed for the crystal growth. The single crystal growth was performed in a 4-mirror furnace equipped with 1000 W halogen lamps. The pulling speed of the rod was 3–5 mm h^−1^. Both rods rotated in opposite direction (speed: ~8 revolutions min^−1^) to ensure efficient mixing of the liquid and a uniform temperature distribution in the molten zone. A pressure of 1.5 bar Ar was used during the crystal growth.

X-ray powder diffraction data on BCGG*x* were collected using a HUBER-Guinier image plate system (Cu ${\mathrm{K}}_{\alpha _1}$, 8° ≤ 2*θ* ≤ 100°). The lattice parameters (Fig. 4a) were obtained from least squares fits to indexed 2*θ* values employing Ge (*a*_Ge_ = 0.5657906 nm) as internal standard.

### Thermal conductivity

Commonly used laser flash methods measure the thermal diffusivity and thus need to be combined with specific heat and density measurements to calculate the thermal conductivity, which typically reduces the accuracy of this technique. By contrast, the 3*ω* method is an ac technique for direct thermal conductivity measurements. During a 3*ω* experiment the sample is heated locally and thus, in contrast to steady-state heat-flow experiments, errors due to radiation at elevated temperatures are reduced to a negligibly low level^24^. Furthermore, this method is insensitive to geometrical errors. This is because the only geometrical parameter entering is the length of a line heater. As it is usually prepared by means of photo or electron-beam lithography and sputtering, its length is very well defined (see below). In fact, the error of our 3*ω* thermal conductivity data, which we estimate to be below 5%, is dominated by uncertainties in the heater resistance and its temperature dependence.

For our studies, the narrow metal line serving as both the heater and the thermometer had a width of 20 μm and a length of 1 mm, with an uncertainly of 1 μm. To avoid electrical contact between heater and sample a thin layer of SiO_2_ was first deposited on the polished sample surfaces by chemical vapor deposition. Then, a 4 nm thick titanium sticking layer and the 64 nm thick gold film were sputtered in an Ardenne LS 320 S sputter system. The heater structures were made by standard optical lithography techniques using a Karl Suess MJB4 mask aligner.

The metal line was heated by an oscillating current at a circular frequency *ω*, which thus leads to a 2*ω* temperature oscillation of both the heater and the sample. Due to the linear temperature dependence of the metallic heater, the 2*ω* temperature oscillation translates into a 3*ω* voltage oscillation, which is detected using a lock-in amplifier (7265, Signal Recovery). Applying the 3*ω* method^24^ to bulk geometry, the measured in-phase temperature oscillation of the heater/thermometer line is expected to be linear in logarithmic frequency *f* as long as the thermal penetration depth is large compared to the heater half width *b* and at least five times smaller than the sample thickness *t* (see boundaries indicated in Fig. 2a). The thermal conductivity *κ* of the material can then be extracted from the slope of the in-phase temperature oscillation Δ*T* vs log *f*.

Prior to the thermal 3*ω* voltage detection, the first harmonic and all related higher harmonics are subtracted from the signal using a carefully gain and phase calibrated active filter^24^ based on a technique that allows to adjust the magnitude and phase of a reference signal as a function of frequency. In this way the main error sources of a 3*ω* experiment, spurious 3*ω* signals arising from harmonic distortions of the involved amplifiers, can be largely eliminated. Using an ultra-precise 100 Ω Z-foil resistor (temperature coefficient ±0.05 ppm °C^−1^, tolerance ±50 ppm; Vishay) as a sample we found the background signal to be negligibly small within the entire frequency range (0.5 Hz to 50 kHz) of the experiment. Using the same resistor, the accuracy of our voltage controlled AC constant current source was found to be ±0.1% within the whole frequency range and for all tested excitations (100 μA to 10 mA). By further measurements on different resistors, load dependences were ruled out.

3*ω* measurements were done in the temperature range between 80 and 330 K. With this setup, we reproduced the thermal conductivity data on single crystalline Ba_8_Ga_16_Ge_30_ of Sales et al.^41^ within the error bar, estimated to only 3% in that work. This precision, which is remarkable for a steady-state experiment, was reached with special radiation shields and specific sample geometries^41,57^.

Below 100 K the data were completed by additional steady-state heat-flow experiments. The phonon thermal conductivities of all investigated materials were calculated by subtracting an electronic contribution, determined using the Wiedemann-Franz law with a constant Lorenz number of *L*_0_ = 2.44 · 10^−8^ WΩK^−2^ and electrical resistivity data measured on the same samples.

### Specific heat

The specific heat was measured with a relaxation-type method using the ^4^He specific heat option of a Physical Property Measurement System (PPMS) from Quantum Design. The addenda was measured separately prior to each sample measurement.

To study the phonon contribution to *C*_p_, first the electronic contribution was determined. At low temperatures, the specific heat can be approximated by *C*_p_/*T* = *γ* + *βT*^2^, where the Sommerfeld coefficient *γ* represents the electron contribution and the *β* parameter quantifies the Debye-like phonon contribution. For BCGG*x*, the data below 3.5 K are very well described by such linear fits (not shown).

Rattling modes, originating from localized oscillations of the guest atoms, can be revealed by analyzing *C*_p_/*T*^3^ vs log*T*. Within such a representation, rattling modes appear as bell-shaped contributions on top of a Debye-like phonon background (not shown).

### Hall effect and electrical resistivity

The electrical resistivity and the Hall coefficient were determined by a standard 6-wire technique using the horizontal rotator option of a Physical Property Measurement System (PPMS) from Quantum Design. Temperature-dependent Hall effect measurements were performed in a magnetic field of 9 T. The Hall resistivity was confirmed to be linear in fields up to 9 T at all temperatures down to 2.5 K. The Hall coefficient was analyzed within a simple one-band model, *R*_H_ = 1/(*ne*).

### Thermal expansion

Measurements of the coefficient of thermal expansion *α*_L_(*T*) = *l*^−1^d*l*/d*T* were carried out by using a high-resolution capacitive dilatometer^58^, which enables the detection of length changes Δ*l* ≥ 10^−2^ Å. Relative length changes were measured along a principle axis of cubic Ba_8_Ga_16_Ge_30_. *α*_L_(*T*) is obtained by numerical differentiation of the Δ*l*(*T*)/*l* data with respect to temperature (Supplementary Note 7).

### Ab initio calculations

Ab initio density functional theory (DFT) simulations were conducted using the Vienna Ab initio Simulation Package (VASP)^59^, applying the projector augmented wave method^60^ and the generalized gradient approximation (GGA) as proposed by Perdew, Burke, and Ernzerhof (PBE)^61^.

In a first step, a fully ordered, cubic 54 atom unit cell of Ba_8_Ga_16_Ge_30_ with Ba at the Wyckoff sites 2*a* and 6*d*, Ge at 6*c* and 24*k*, and Ga at 16*i* was investigated, using a 5 × 5 × 5 *k*-point mesh and a plane wave cutoff of 500 eV. For a fixed lattice constant of 10.74 Å, corresponding to the experimental value, the atomic positions were relaxed. To test the impact of a more realistic Ga/Ge distribution^22^, we have in addition performed selected calculations for a disordered structure (see Supplementary Notes 9 and 11, and Supplementary Fig. 5). After reaching convergence (residual forces of less than 10^−3^ eV Å^−1^), symmetry non-equivalent displacements were introduced into the relaxed structure of both the ordered and the disordered (see Supplementary Table 2) unit cell. For displacements of 0.02 Å, the restoring forces were determined, again by using the VASP code with the above described settings. From the obtained forces, the dynamical matrix was extracted and the ALAMODE code^23,62^ was used to determine the phonon DOS and the specific heat (Supplementary Fig. 1, main parts and insets) in the harmonic approximation.

The thermal expansion coefficient for both the ordered and disordered structure was determined within the quasi-harmonic approximation. For this purpose, the ground state structures and energies were determined for a series of unit cells with lattice parameters, corresponding to both decreased and increased cell volumes. For each of these volumes the dynamical matrix was then obtained in the same way as described above for the experimental volume. By using the phonopy code^63^ the free energy was determined at the given volume and as a function of temperature. The free energy as a function of volume for a given temperature was then fitted to the Vinet equation of state, again using the phonopy code. Finally, from the free energy minima at a given temperature, the corresponding equilibrium volume at this temperature can be extracted, which then allows to access the thermal expansion coefficient. The ab initio thermal expansion curves are slightly rescaled to match the high-temperature data (Supplementary Fig. 2).

The mode-specific Grüneisen parameter was obtained for both the ordered and the disordered model structures (Supplementary Fig. 5e). Again, the lattice parameter of the equilibrium structure was fixed to the experimental value of 10.74 Å and the dynamical matrix was determined as discussed above. To evaluate the behavior of the vibrational frequencies with respect to a volume change, the dynamical matrix was also determined for increased and decreased lattice parameter (±0.5%). Relating the frequency and volume change of a phonon mode allows then to extract the mode-specific Grüneisen parameter. This procedure is also available within the phonopy code^63^.

The lattice thermal conductivity of the fully ordered structure was calculated from ab initio results for the anharmonic force constants and by applying the relaxation time approximation, as implemented in the ALAMODE code^23,62^ (see Supplementary Notes 10 and 11 for details). Our calculations reproduce the temperature dependence of the lattice thermal conductivity obtained by Tadano et al.^23^ for the same structure model accurately, and the absolute value of the lattice thermal conductivity within a factor of 1.1 (which is most likely due to slightly different lattice constant used in both calculations). Expanding the range of anharmonic interactions from nearest to next-nearest neighbor shells leaves the temperature dependence largely unaffected (for Fig. 3d these results are used). The same is expected if the anharmonic force constants are allowed to vary (within ranges compatible with experiments) with temperature (Supplementary Note 11). Finally, the temperature dependence of the calculated thermal conductivity is shown to be robust against disorder within the Ga-Ge framework (for details see Supplementary Notes 9 and 11 and Supplementary Fig. 5f).

### Code availability

Licenses for VASP are available on the VASP website (https://www.vasp.at/). The ALAMODE (https://alamode.readthedocs.io) and phonopy (https://atztogo.github.io/phonopy/) packages are open source.

## Supplementary information

Supplementary Information

## Data Availability

The data sets generated and/or analyzed during the current study are available from the corresponding author on reasonable request.
