# Supplementary material for: Kondo-like phonon scattering in thermoelectric clathrates
Source: Nat Commun. 2019 Feb 21;10:887. doi: 10.1038/s41467-019-08685-1 (PMC6385256; doi:10.1038/s41467-019-08685-1)
Supplement: Supplementary file 1 — Supplementary Information [file 41467_2019_8685_MOESM1_ESM.pdf]

Supplementary Information for

Kondo-like phonon scattering in thermoelectric clathrates

Ikeda et al.

Supplementary Discussion: Supplementary Notes 1 - 11

Supplementary Tables 1, 2

Supplementary Figures 1 - 5

Supplementary References

Supplementary Discussion

Supplementary Note 1: Modified Callaway model

The Callaway model[1] is a phenomenological treatment of the phonon thermal conductivity of solids. It is derived from the Boltzmann equation for an isotropic crystal with dispersionless acoustic phonon branches, assuming that all phonon scattering processes can be represented by frequency-dependent relaxation times. Both resistive three-phonon Umklapp scattering processes and total momentum conserving normal processes are considered. Its simplicity and early success in describing simple materials such as Germanium made it a popular choice to model experimental thermal conductivity data, even for more complex materials.

To model the low-temperature thermal conductivity of type-I clathrates we propose here a modified form of the Callaway model, namely

$$\begin{aligned}\kappa_{\mathrm{ph}} &= \frac{k_{\mathrm{B}}^4 \cdot T^3}{2 \cdot \pi^2 \cdot v_{\mathrm{s}} \cdot \hbar^3} \cdot \left[\int_0^{\frac{\Theta_{\mathrm{E}}}{T}} \tau \cdot \frac{x^4 \cdot e^x}{(e^x - 1)^2} \cdot dx + \frac{I_1^2}{I_2} \right] \quad , \\ \text{with} \quad I_1 &= \int_0^{\frac{\Theta_{\mathrm{E}}}{T}} \frac{\tau}{\tau_{\mathrm{N}}} \cdot \frac{x^4 \cdot e^x}{(e^x - 1)^2} \cdot dx \quad , \\ I_2 &= \int_0^{\frac{\Theta_{\mathrm{E}}}{T}} \frac{1}{\tau_{\mathrm{N}}} \cdot \left(1 - \frac{\tau}{\tau_{\mathrm{N}}}\right) \cdot \frac{x^4 \cdot e^x}{(e^x - 1)^2} \cdot dx \quad , \quad \text{and} \\ \tau^{-1} &= \tau_{\mathrm{N}}^{-1} + \tau_{\mathrm{D}}^{-1} + \tau_{\mathrm{B}}^{-1} + \tau_{\mathrm{U}}^{-1} + \tau_{\text{ph-el}}^{-1} \quad .\end{aligned} \tag{1}$$

It differs from the original Callaway model only by the substitution of the Debye temperature Θ_{D} by the characteristic temperature of the lowest-lying Einstein contribution Θ_{E}. This approximation effectively cuts off all phonon modes above the lowest-lying optical mode, which is justified by the significantly smaller group velocities of the higher-lying modes[2,3]. At temperatures below $\Theta_{\mathrm{E}}/2$, where we performed quantitative analyses (Fig. 3b, Fig. 4d, e of main part), errors arising from this approximation are negligible. The empirical scaling of the intrinsic phonon thermal conductivities of a large number of clathrates with only $v_{\mathrm{s}}\Theta_{\mathrm{E}}$ observed at 300 K (Fig. 5 of main part) further supports this approximation because Θ_{E} does not contain any information on the phonon spectrum above Θ_{E}.

Normal processes are usually taken into account by

$$\tau_{\mathrm{N}}^{-1} = N\omega^a T^b \quad . \tag{2}$$

However, for complex crystal structures or materials including a high defect concentration these processes have been shown to be negligible[4] permitting us to omit the corresponding

I_1^2/I_2 term in Supplementary Equation 1, in our analyses. At low temperatures, we describe defect scattering by Rayleigh scattering[5]

$$\tau_{\mathrm{D}}^{-1} = D \cdot \omega^4 \tag{3}$$

and boundary scattering by the frequency independent scattering rate[5]

$$\tau_{\mathrm{B}}^{-1} = B \quad . \tag{4}$$

In analogy with the expression for the scattering rate due to three phonon Umklapp scattering within the Debye approximation[5]

$$\tau_{\mathrm{U}}^{-1} \propto \omega^2 e^{-\frac{\Theta_{\mathrm{D}}}{2T}} \left[1 + 6\frac{T}{\Theta_{\mathrm{D}}} + 24\left(\frac{T}{\Theta_{\mathrm{D}}}\right)^2 + 48\left(\frac{T}{\Theta_{\mathrm{D}}}\right)^3\right] \quad \text{for} \quad T < \Theta_{\mathrm{D}} \quad , \tag{5}$$

we derived[6]

$$\tau_{\mathrm{U}}^{-1} \propto \frac{\omega^2}{\alpha^2} e^{-\frac{\Theta_{\mathrm{E}}}{\alpha T}} \left[1 + 2\frac{\alpha T}{\Theta_{\mathrm{E}}} + 2\left(\frac{\alpha T}{\Theta_{\mathrm{E}}}\right)^2\right] \quad \text{for} \quad T < \Theta_{\mathrm{E}} \tag{6}$$

for acoustic phonons scattering off a low-lying flat optical phonon mode with energy $k_{\mathrm{B}}\Theta_{\mathrm{E}}$. The exponential term of τ_{U}^{-1} derived within the Debye approximation is scaled by Θ_{D}. By contrast, in the model proposed here, the Einstein temperature Θ_{E} determines the Umklapp scattering rate. As in the original form[5], the parameter α scales the energy of the second phonon to be able to participate in three phonon Umklapp scattering. The upper limit of α is determined by the dispersion of the acoustic branch. α values around 2.3 obtained from fitting κ_{ph} of the BCGG series (not shown) are reasonable[5,7].

An alternative relation for τ_{U}^{-1} of type-I clathrates and related materials is obtained[8] by substituting Θ_{D} by Θ_{E} in the empirical expression[9]

$$\tau_{\mathrm{U}}^{-1} = U\omega^{\alpha} e^{-\frac{\Theta_{\mathrm{D}}}{bT}} \left(\frac{T}{\Theta_{\mathrm{D}}}\right)^{\beta} \quad . \tag{7}$$

Phonon-electron scattering is taken into account by the expression[10]

$$\tau_{\text{ph-el}}^{-1} = \frac{\pi n m^* v_{\mathrm{F}} \omega}{6\rho v_{\mathrm{s}}} \quad \text{with} \quad q < 2k_{\mathrm{F}} \quad \text{and} \quad q l_{\mathrm{e}} >> 1 \quad , \tag{8}$$

where n is the charge carrier concentration, m^* is the effective electron mass, v_{F} is the Fermi velocity, ρ is the mass density, and l_{e} is the electron mean free path.

Finally we suggest a redefinition of Cahill's minimum phonon thermal conductivity[11] to the new form

$$\kappa_{\mathrm{ph}}^{\mathrm{min}} = \frac{k_{\mathrm{B}}^4 \cdot T^3}{2 \cdot \pi^2 \cdot v_{\mathrm{g}} \cdot \hbar^3} \cdot \int_0^{\frac{\Theta_{\mathrm{E}}}{T}} \frac{x^3 \cdot e^x}{(e^x - 1)^2} \cdot dx \quad . \tag{9}$$

Cutting off phonon modes above Θ_E is justified by the same arguments as in Supplementary Equation 1. A direct confirmation of the need for a new κ_{ph}^{min} limit is the experimental observation of the violation of the original (Cahill) limit (see Supplementary Discussion S2).

Supplementary Note 2: Materials

Figure 3b right compares the phonon thermal conductivity of the single crystalline clathrate BCGG1.0 with elemental Ge. Both materials possess a similar Debye temperature (304 K for BCGG1.0, 362 K for Ge, Ref. 12). In both cases, phonon-electron scattering and boundary scattering are expected to be negligible. In contrast to the complex structure of BCGG1.0 built up of atoms with severely different masses, single crystalline Ge typically exhibits few defects and thus a very high thermal conductivity. Therefore, the phonon thermal conductivity of an electron-irradiated and post-annealed Ge sample (Ge II annealed at 77 K, Ref. 12) was chosen for comparison.

Figure 4e compares the phonon thermal conductivity of two type-I clathrate single crystals with nominal composition $Ba_8Ga_{16}Ge_{30}$. Phonon-electron scattering can only occur for phonons with a wave vector q smaller than twice the Fermi wave vector k_F. Using the charge carrier concentration $n = 0.84 \cdot 10^{27}\,\mathrm{m}^{-3}$ and the sound velocity calculated from the Debye temperature $\Theta_D = 324\,\mathrm{K}$ reported for crystal II of Ref. 13 we estimate that phonon-electron scattering should occur below 140 K.

For a few type-I clathrates[14,15] and skutterudites[16,17], phonon thermal conductivities have been reported that violate the Cahill κ_{ph}^{min} limit, even though this was not explicitly stated in these works. For nanostructured $Ba_8Cu_xSi_{46-x}$ ($x = 3.6, 3.8$) prepared by ballmilling and hotpressing $\kappa_{ph}(800\,\mathrm{K}) = 0.2\,\mathrm{W\,K^{-1}m^{-1}}$ was found[14]. With the reported Debye temperature $\Theta_D = 420\,\mathrm{K}$ we calculate a Cahill κ_{ph}^{min} value of $0.85\,\mathrm{W\,K^{-1}m^{-1}}$. A less drastic violation seems to occur for $K_8Ga_8Si_{38}$ with $\kappa_{ph}(300\,\mathrm{K}) = 0.5\,\mathrm{W\,K^{-1}m^{-1}}$ (Ref. 15). In the absence of reported Θ_D values we estimate a Cahill κ_{ph}^{min} value of $0.7\,\mathrm{W\,K^{-1}m^{-1}}$ as lower boundary by using $\Theta_D = 360\,\mathrm{K}$ of the heavier-element Si-based clathrates La-BAS and Ce-BAS (Supplementary Table 1) as lower Θ_D boundary. Likewise, also the high-pressure torsion-treated skutterudites $DD_xFe_3CoSb_{12}$ with $x = 0.6$ (Ref. 16) and $x = 0.68$ (Ref. 17) show ultralow phonon thermal conductivities that appear to violate the respective Cahill κ_{ph}^{min} limits.

Supplementary Fig. 1a shows the specific heat at constant volume (C_V) of Si versus temperature. The data were calculated from experimental specific heat values taken at constant

pressure (C_p, Ref. 18) and the temperature dependent volumetric thermal expansion coefficient (α_V, Ref. 19) using the expression $C_\mathrm{p} - C_\mathrm{V} = \alpha_\mathrm{V}^2 \cdot T \cdot V_\mathrm{mol} \cdot B$, where V_mol is the molar volume and $B = 97.8\,\mathrm{GPa}$ is the bulk modulus of Si (Ref. 20). For $Ba_8Ga_{16}Ge_{30}$ (BGG, Supplementary Fig. 1b), the thermal expansion coefficient was taken from Ref. 21 and the bulk modulus from Ref. 22.

Supplementary Note 3: Filling of vacancies by Ga in $\mathbf{Ba_8Cu_{4.8}Ge_{41.2-x-y}\square_yGa_x}$

Here we show that with increasing Ga content x in $Ba_8Cu_{4.8}Ge_{41.2-x-y}\square_yGa_x$ the content y of vacancies $\square$ is successively reduced. We assume that, as in related systems[23–25], both Cu and the vacancy occupy the $6c$ site (Fig. 1a,b). With the multiplicity 6 of this site and 4.8 Cu atoms this limits the maximum amount of vacancies to 1.2 per unit cell. The linear dependence of the lattice parameter a on the Ga content x with the slope $\Delta a/\Delta x = 4.8\,\mathrm{m\AA}$ (Fig. 4a) suggests that Ga successively fills the vacancies and that we thus have $y = 1.2 - x$. In detail, this slope is well accounted for by a substitution of Ge for $\square$ with $\Delta a/\Delta x = 3.46\,\mathrm{m\AA}$ (Ref. 23) in a first step, and by a substitution of Ga for Ge with $\Delta a/\Delta x = 1.13\,\mathrm{m\AA}$ (blue line in Fig. 4a) in a second step. Both steps together yield a slope of $4.59\,\mathrm{m\AA}$ $(= 3.46 + 1.13)$, in good agreement with experiment (red line in Fig. 4a).

The filling of the vacancies by Ga is corroborated by our low-temperature specific heat C_p and electrical transport (resistivity ρ and Hall coefficient R_H) measurements. Whereas the prefactor β of the phonon T^3 term of $C_\mathrm{p}(T)$ increases strongly with the rate $\Delta\beta/\Delta x \approx 30$ (Fig. 4b), the electronic Sommerfeld coefficient γ (Fig. 4b) and the charge carrier concentration n (Fig. 4c bottom) are essentially independent of x. This discards Ga substituting either Cu or Ge which, according to the Zintl-Klemm concept[26] (see Supplementary Discussion S4), would change the charge carrier concentration by +2 or -1 electron per formula unit, respectively, and suggests that the formal charge of the vacancy is -1, just as that of Ga. The observed increase of the charge carrier mobility $\mu_\mathrm{H} = R_\mathrm{H}/\rho$ with increasing Ga content (Fig. 4c top and bottom) strongly supports the picture of vacancy filling because in a Ge-based clathrate electrons are expected to scatter much more strongly from vacancies than from Ga.

Supplementary Note 4: Zintl-Klemm concept

For the BCGG series a nearly temperature and sample independent charge carrier concentration n was found. According to the Zintl concept, Ba is expected to donate 2 electrons

to the host framework. In a first approximation, the tetrahedrally coordinated host atoms can be considered to form covalent bonds. The formal charge of a Ge/Ga/Cu atom within a type-I clathrate host framework is expected to be close to 0/-1/-3. Therefore, one Ga atom substituting for Ge (Cu) would change the charge carrier concentration by -1 (+2) electrons per formula unit. For the BCGG series, the variable amount of Ga incorporated into the host framework leaves the charge carrier concentration nearly unchanged. Therefore, a substitution of Ga for vacancies with a formal charge of -1 is the most likely scenario.

Supplementary Note 5: Specific heat analysis

The phonon specific heat of type-I clathrates is typically described by the sum of a Debye contribution and two Einstein contributions,

$$C_{\mathrm{p}} = 9N_{\mathrm{D}}R\left(\frac{T}{\Theta_{\mathrm{D}}}\right)^3\int_0^{\Theta_{\mathrm{D}}/T}\frac{x^4e^x}{(e^x-1)^2}dx + \sum_{\mathrm{i}=1}^{2}p_{\mathrm{i}}N_{\mathrm{Ei}}R\left(\frac{\Theta_{\mathrm{Ei}}}{T}\right)^2\frac{e^{\Theta_{\mathrm{Ei}}/T}}{(e^{\Theta_{\mathrm{Ei}}/T}-1)^2} \quad , \tag{10}$$

where $x = \hbar\omega(k_{\mathrm{B}}T)$ and N_{D} and N_{Ei} are the numbers of Debye and Einstein oscillators per formula unit. Θ_{D}, Θ_{Ei}, and p_{i} are the Debye temperature, Einstein temperatures, and the number of degrees of freedom related to the i-th vibrational mode of the guest atoms, respectively, and R is the gas constant. In the fitting procedure we fixed $p_1 = p_2 = 3$ and $N_{\mathrm{D}} + \sum N_{\mathrm{Ei}} = 54$. When presented as $\frac{C_{\mathrm{p}}}{T^3}$ vs $\log T$, Einstein contributions typically have a bell-shaped appearance on top of a Debye background from which the corresponding Einstein temperatures can be extracted by fitting with Supplementary Equation 10.

Below 3.5 K, the specific heat of all samples analyzed here has the form $C_{\mathrm{p}}/T = \gamma + \beta T^2$, which was used to extract the sound velocity v_{s}. The number of contributing atoms was assumed to be 54. When inelastic neutron scattering data were available, v_{s} was extracted from these using the relation $v_{\mathrm{s}} = [1/3\cdot((1/v_{\mathrm{L}})^3 + 2\cdot(1/v_{\mathrm{T}})^3)]^{-1/3}$, where v_{T} and v_{L} are the initial slopes of the transverse and longitudinal acoustic phonon branches, respectively. The agreement between both methods is satisfying.

Supplementary Note 6: Theoretical calculation of the specific heat

The vibrational properties of $Ba_8Ga_{16}Ge_{30}$ and Si were computed with *ab initio* density functional theory (Methods). The specific heat at constant volume was calculated from the phonon density of states $D(\omega)$, shown in Supplementary Fig. 1 insets, via

$$C_{\mathrm{V}}(T) = \frac{\partial}{\partial T}\int_0^{\infty} d\omega D(\omega)\left[\frac{1}{e^{\frac{\hbar\omega}{k_{\mathrm{B}}T}}-1}\right](\hbar\omega + \frac{1}{2}) \quad . \tag{11}$$

Supplementary Note 7: Experimental determination of the thermal expansion

The linear coefficient of thermal expansion $\alpha_{\rm L}(T)$ of single crystalline $Ba_8Ga_{16}Ge_{30}$ was determined from the experimentally revealed $\Delta l(T)/l$ data, with $\Delta l(T) = l(T) - l(200\,{\rm K})$, by numerical differentiation with respect to temperature. For that purpose the $\Delta l(T)/l$ data were divided into equidistant intervals of width $\Delta T = 0.13\,{\rm K}$, in each of which the mean slope was determined from linear regression. The experiment was carried out on a single crystal of length $l = 1.6\,{\rm mm}$ along a principal axis of the cubic structure for which the volume expansion coefficient $\alpha_{\rm V}$ is given by $\alpha_{\rm V} = 3 \cdot \alpha_{\rm L}$. Measurements were performed from 4.5 K up to 200 K upon increasing the temperature at a slow rate of $1.5\,{\rm K\,h^{-1}}$ to ensure thermal equilibrium. At low temperatures, the data are well described by $\alpha_{\rm V}/T = a + bT^2$, with a negligibly small electronic contribution a (not shown). Thus, $\alpha_{\rm L}(T)$ is dominated by the phonon contribution.

Supplementary Note 8: Phonon Kondo effect

The Kondo problem has a 50-year-old history of highly active research. It has been studied in settings ranging from bulk materials, to surfaces, 2-dimensional materials, mesoscopic systems, and ultracold atoms in optical lattices. Originally it was formulated for free conduction electrons interacting with spin-1/2 local magnetic moments[27–29]. However, Kondo physics is a very broad phenomenon, with only a few fundamental requirements, most notably the non-commutative scattering of an extended wave off a localized entity with internal degrees of freedom. In addition to magnetic moments, the localized entities may take the form of orbital[30–32], charge[33], and local vibrational degrees of freedom[34–37]. The extended wave component can also take various forms, in addition to conduction electrons in simple metals, it may also arise from fermions with pseudo-gapped electronic densities of states[38], Dirac particles[39,40], and bosonic baths[41,42]. To the best of our knowledge Kondo physics has, however, never been considered in an entirely phononic context.

In the following we give an experimentalist's sketch of how the basic physical picture of a Kondo interaction between rattling modes and the transverse acoustic phonons of a type-I clathrate, described in the main text, might be translated into a Kondo-type Hamiltonian.

The two degenerate Einstein modes associated with the two rattling directions ($\alpha = 1, 2$)

of the guest atoms in the large cages are described as

$$H_0 = \sum_{\alpha=1,2} \hbar\omega_{\rm E}(b_\alpha^\dagger b_\alpha + \frac{1}{2}) + \frac{U}{2}(b_1^\dagger b_1 + b_2^\dagger b_2 - N_b)^2 \quad , \tag{12}$$

where $b_\alpha^\dagger$ and b_α ($\alpha = 1, 2$) are the boson creation and annihilation operators, N_b is an effective boson number, and U is the interaction energy. We can absorb the first term into the second, writing

$$H_0 = \frac{U}{2}(n_1 + n_2 - N_b^*)^2 + \hbar\omega_{\rm E}(1 + \frac{\hbar\omega_{\rm E}}{2U} + N_b^*) \quad , \tag{13}$$

where

$$N_b^* = N_b - \frac{\hbar\omega_E}{U} \tag{14}$$

and $b_\alpha^\dagger b_\alpha = n_\alpha$. Provided this quantity remains positive, then the ground state energy is minimized at $n_1 + n_2 = N_b^*$, and the energy of the states with one more, or one less phonon is $U/2$ higher. In this way, our local Hamiltonian has the effect of inducing a "phonon blockade", the rattler analog of the Coulomb blockade of the spin Kondo effect, in which the states with N_b^* phonons are degenerate, and split off beneath all states with larger, or smaller numbers of bosons. The Hamiltonian also ensures that there is an effective SU(2) degeneracy between the 1 and 2 bosons. However, it may well be that other formulations can be found that represent a phonon Kondo effect more elegantly.

The hybridization of the Einstein modes with the acoustic modes is described by

$$H_1 = \sum_{\mathbf{q},\alpha=1,2} V_{\mathbf{q}}(b_{\mathbf{q},\alpha} + b_{-\mathbf{q},\alpha}^\dagger)(b_\alpha + b_\alpha^\dagger) \tag{15}$$

where $V_{\mathbf{q}}$ is the hybridization strength. It does not conserve the boson number. This will produce additional Kondo terms in the Schrieffer-Wolff transformation, in which the number of acoustic bosons changes by ± 2.

Finally, the acoustic modes themselves are described by

$$H_2 = \sum_{\mathbf{q},\alpha=1,2} \hbar\omega_{\mathbf{q},\alpha}(b_{\mathbf{q},\alpha}^\dagger b_{\mathbf{q},\alpha} + \frac{1}{2}) \quad , \tag{16}$$

where $\mathbf{q}$ is the wave vector and α the polarization of an acoustic phonon. The total Hamiltonian of the interacting acoustic and rattling phonon system thus reads

$$H = H_0 + H_1 + H_2 \quad , \tag{17}$$

which resembles an Anderson impurity model[28].

To map this Hamiltonian directly to a spin Kondo Hamiltonian (Schrieffer-Wolff transformation[28,29]) we note that the two-fold degeneracy of the rattler modes ($\alpha = 1, 2$) resembles a spin, described in a Schwinger boson representation[43–45] as

$$S_z = \frac{1}{2}(b_1^\dagger b_1 - b_2^\dagger b_2) \quad , \tag{18}$$

where

$$S = \frac{1}{2}(b_1^\dagger b_1 + b_2^\dagger b_2) \quad . \tag{19}$$

Interestingly, this means that the effective spin of the rattler is given by the Bose-Einstein function

$$2S = N_b = 1/(e^{\hbar\omega_{\mathrm{E}}/(k_{\mathrm{B}}T)} - 1) \tag{20}$$

and will thus grow with $k_{\mathrm{B}}T/(\hbar\omega_{\mathrm{E}})$ at high temperatures. This distinguishes the all phononic Kondo effect from the usual spin Kondo effect in a simple metal, where the interaction does not disappear at $T = 0$. By contrast, it is similar to the Kondo effect in pseudo-gapped Fermi systems[38] and in particular in fermionic systems with linear dispersion like graphene and 3-dimensional Dirac semimetals. Here the electronic density of states vanishes at the Fermi level and thus Kondo screening is expected to disappear below the Kondo temperature[39]. Interestingly, a $-\ln T$ dependence of the electrical resistivity is experimentally observed in defect-graphene in a rather extended temperature range above some cutoff temperature[40].

The acoustic phonon modes apparently have the ability to switch the polarization of the rattler, which can be visualized as follows: If an acoustic phonon of polarization 1 arrives at a cage with an atom rattling in polarization 2, it will be absorbed in the cage in the form of a distortion of the cage along 1, corresponding to an excited state (represented by the Hubbard term in Supplementary Equation 12). This may lead to a change of the rattling direction ($2 \to 1$), and the subsequent emission of an acoustic phonon of changed polarization ($1 \to 2$). Such a process is governed by the "antiferromagnetic" exchange interaction

$$J \sim \frac{V^2}{U} \tag{21}$$

where, for simplicity, we have assumed the hybridization to be wave vector independent. This leads to the well-known Kondo term

$$H_{\mathrm{K}} \sim J\mathbf{s}(0) \cdot 2\mathbf{S} \quad , \tag{22}$$

where $s(0)$ represents the effective pseudospin

$$s(0) = \phi^\dagger_\alpha \sigma_{\alpha\beta} \phi_\beta, \tag{23}$$

of the acoustic phonons at the position of the local pseudospin $2S$, $\sigma_{\alpha\beta}$ are the Pauli matrices, and

$$\phi_\alpha = \sum_{\mathbf{q}} g_{\mathbf{q},\alpha}(b_{\mathbf{q},\alpha} + b^\dagger_{-\mathbf{q},\alpha}) \tag{24}$$

are the displacements of the acoustic modes that couple linearly to the rattler modes.

To treat this or equivalent formulations of the all phononic Kondo effect we believe to be realized in type-I clathrates and derive its physical properties for a realistic phonon density of states as function of the coupling strength is a task for future theoretical work. However, in analogy with related models (see above), it seems likely that archetype Kondo features such as the observed low-temperature ($-\ln T$-like) increase of the thermal resistance would indeed result from such a model, at least in a limited temperature range.

Supplementary Note 9: Effects of site disorder on DFT results

For $Ba_8Ga_{16}Ge_{30}$, previous DFT calculations used a fully ordered model with Ga occupying only the $16i$ site[46]. However, a comparison of DFT calculations for the binary type-I clathrate K_8Si_{46} and its ternary variant $K_8Al_8Si_{38}$, obtained by randomly substituting 8 Si atoms by Al, revealed distinct differences in the phonon dispersion and density of states[47]. Thus, we have also investigated possible disorder effects in $Ba_8Ga_{16}Ge_{30}$.

To mimic the experimentally observed Ga/Ge occupation numbers[21], a disordered structure was created (for structural details see Supplementary Table 2) using the special quasi-random structure (SQS) approach[48]. It distributes the atomic species on the sites of a unit cell by a Monte Carlo algorithm, such that their correlation functions match those of an infinite random distribution as closely as possible. With this SQS approach, we were able to obtain the phonon dispersion, specific heat, thermal expansion, mode-averaged and mode-specific Grüneisen parameter, as well as the thermal conductivity (for details on the calculations, see Supplementary Discussion S11). A comparison of the results with those for the fully ordered structure model reveals that all properties are qualitatively similar (Supplementary Fig. 5); most notably, the temperature dependences are robust against the introduced disorder (compare scaled curves indicated by dashed lines in Supplementary Fig. 5c,d,f). Also the mode-specific Grüneisen parameter is similar for both models. The

larger spread in the low-energy data for the disordered model is attributed to the reduced symmetry of this model. Averaging over modes in the acoustic regime results in a somewhat higher Γ^{acoustic} value for the disordered model (Supplementary Fig. 5e), in agreement with the reduced absolute value of the thermal conductivity (Supplementary Fig. 5f). However, importantly, the temperature dependence of the thermal conductivity is essentially unaffected by the disorder.

That the effect of disorder is much smaller in $Ba_8Ga_{16}Ge_{30}$ than in $K_8Al_8Si_{38}$ might be due to the fact that, while the mass difference between Al and Si and between Ga and Ge is similar (4% in both cases), the covalent radii of Al and Si differ considerably (by 8.6%) but are essentially identical for Ga and Ge. Nevertheless, as the disordered model seems more appropriate for the real material, in Fig. 3c of the manuscript we compare the measured specific heat and thermal expansion to calculations for the disordered model. As to the thermal conductivity, our calculations on the disordered model should be checked for convergence with respect to an increasing range of anharmonic interactions; however, such calculations are computationally extremely demanding and currently unavailable. Therefore, in Fig. 3d of the manuscript, the experimental results are compared to calculations for the ordered structure. In view of the robustness against disorder of the temperature dependence of (i) the thermal conductivity for nearest neighbor anharmonic interactions (Supplementary Fig. 5f), (ii) the thermal expansion, and (iii) the Grüneisen parameter, the latter two being well-known measures of phonon anharmonicity, we expect differences in further advanced calculations of the thermal conductivity to also be small, most definitely not of the order of 300% as the discrepancy between experiment and computation at room temperature.

Supplementary Note 10: Effects of the exchange-correlation functional on the thermal conductivity

To also rule out the possibility that the observed strong underestimation of the room-temperature lattice thermal conductivity is an artifact of the chosen exchange-correlation functional (PBE) in our DFT calculations, we have conducted further calculations using different exchange-correlation functionals. As expected from other studies, we indeed find calculations with the LDA functional yielding a lower lattice thermal conductivity than our PBE calculations. To go one step further, we additionally have investigated the lattice thermal conductivity using the SCAN functional. Here, we also find a lower lattice thermal

conductivity than for the PBE calculations. Thus, other functionals just further increase the discrepancy between experiment and computation.

Supplementary Note 11: Calculating the lattice thermal conductivity

For the calculation of the lattice thermal conductivity it is necessary to determine the anharmonic interactions (third order force constants). These are obtained by displacing two atoms at a time out of their equilibrium positions (we have used displacements of 0.04 Å). For the resulting configurations (symmetry considerations significantly reduce the number of different configurations), the restoring forces are calculated as described in the main text.

As a starting point for our calculations of the lattice thermal conductivity, we have used the alamode code[46,49] with the same setup as proposed by Tadano et al.[46]. To ensure the convergence of the published results[46], obtained for a $6 \times 6 \times 6$ q-point mesh, we have repeated our calculations for different q-point meshes ($8 \times 8 \times 8$ and $10 \times 10 \times 10$). The thermal conductivities for all three cases were found to be in good agreement (the results for the $6 \times 6 \times 6$ mesh agree within 10% with the values found for the $10 \times 10 \times 10$ mesh). Most importantly, all calculations yield essentially the same temperature dependence. In a next step, we increased the range of anharmonic interactions from nearest to next-nearest neighbor interactions. While this increased the absolute value of the thermal conductivity (by about 60%), the temperature dependence was again found to be unchanged. The final setup used for all analyses throughout the manuscript comprised a $10 \times 10 \times 10$ q-point mesh and next-nearest neighbor anharmonic interactions. In addition to Umklapp scattering, we also included boundary scattering on a length scale of 2000 nm (to account for the experimental thermal conductivity at low temperatures). All calculations were done within the relaxation time approximation (RTA) as implemented in the alamode code[46,49], which usually works well for materials with low lattice thermal conductivity. In fact, a recent study for Ba_8Si_{46} has shown that the full solution of the Boltzmann transport equation yields essentially the same result as the RTA (Ref. 50). Likewise, taking the temperature dependence of the interatomic force constants into account is expected to have only minor effects on the temperature dependence of the thermal conductivity of type-I clathrates. In a recent DFT study[51], that vastly overestimates the temperature effect as evidenced by comparison with experiments[52,53], a $\kappa \sim T^{-0.7}$ dependence was found, which is still at odds with the experimental thermal conductivity.

Finally, to show that the temperature dependence of the calculated thermal conductivity is robust against disorder, we have also performed thermal conductivity calculations for disordered $Ba_8Ga_{16}Ge_{30}$ (see Supplementary Discussion S9 for the disorder model). Due to the huge set of displacements that would have to be calculated, it is impossible to determine the anharmonic force constants as described above. We have thus chosen a different approach: We have run *ab initio* molecular dynamics simulations (at 500 K, 10.000 steps, 2 fs) to obtain 90 input configurations for fitting the anharmonic interactions. In these configurations, additional random displacements were introduced such that, finally, a set of roughly 400 configurations with random displacements was obtained. For these configurations, the corresponding forces were calculated and finally the anharmonic interactions were fitted. These calculations were done taking into account nearest neighbor anharmonic interactions. Though the absolute value of the thermal conductivity of disordered $Ba_8Ga_{16}Ge_{30}$ is lower than in the ordered case, the temperature dependence is essentially unaffected by the disorder (Supplementary Fig. 5f).

Supplementary Tables

Abbreviation	Composition	Reference
BCGG0.0	$Ba_8Cu_{4.8}Ge_{40}\square_{1.2}$	
BCGG0.2	$Ba_8Cu_{4.8}Ge_{40}Ga_{0.2}\square_{1.0}$	
BCGG0.5	$Ba_8Cu_{4.8}Ge_{40}Ga_{0.5}\square_{0.7}$	
BCGG1.0	$Ba_8Cu_{4.8}Ge_{40}Ga_{1.0}\square_{0.2}$	
BNG	$Ba_8Ni_{3.5}Ge_{42.1}\square_{0.4}$	[23]
BAG	$Ba_8Au_{5.25}Ge_{40.3}\square_{0.45}$	[24,54]
BAgG	$Ba_8Ag_{4.1}Ge_{41.4}\square_{0.5}$	[25]
La-BAS	$La_{1.23}Ba_{6.99}Au_{5.91}Si_{39.87}$	[55]
Ce-BAS	$Ce_{1.06}Ba_{6.91}Au_{5.56}Si_{40.47}$	[55]
BGG	$Ba_8Ga_{16}Ge_{30}$	[52,56]
SGG	$Sr_8Ga_{16}Ge_{30}$	[56,57]
EGG	$Eu_8Ga_{16}Ge_{30}$	[56,58]
BGSn	$Ba_8Ga_{16}Sn_{30}$ (n-type)	[59–61]
Xe hydrate	$Xe{\cdot}6.2H_2O$	[62–64]
CMSS	$Cu_{10.6}Mn_{1.4}Sb_4S_{13}$	[65]

Supplementary Table 1: Materials obeying universal scaling. Type-I clathrate single crystals synthesized for this work (BCGGx, BGG) and materials used for comparison. All intermetallic clathrates except the large-grain polycrystal BAgG are single crystals. The other materials are polycrystals.

Atom	x	y	z
Ga1	0.4975264989695782	0.2505663337067959	0.9992496843678507
Ga2	0.9998920218899980	0.5005665009056267	0.2460261578088667
Ga3	0.2495784802091952	0.0004068320168955	0.4959776641127093
Ga4	0.7447794238830292	0.0022870861176575	0.4994553078971444
Ga5	0.1818660097771889	0.8149481164101957	0.1845553666057027
Ga6	0.8159870134144092	0.1840168286893603	0.8138216352311991
Ga7	0.3129445795085927	0.3138743476334949	0.6808105497837265
Ga8	0.6894865024829355	0.6832641429864595	0.3205249637536032
Ga9	0.1150091812861667	0.9978668271669152	0.3090892823945013
Ga10	0.3110073711563373	0.1157647360317709	0.0039467894125918
Ga11	0.3094876030057422	0.8814796484006135	0.0044172136363994
Ga12	0.6922431930331699	0.8817166118168954	0.0008457814288131
Ga13	0.4928356812357650	0.3828159925516351	0.8103440766261054
Ga14	0.1925922479171963	0.4993165452623339	0.6151002061529589
Ga15	0.4987066084392095	0.6160084998139452	0.8110877488418893
Ga16	0.6178074800108676	0.1923835477763470	0.5014987380709244
Ge1	0.5003469852878626	0.7511381399658155	0.9990963957684287
Ge2	0.0030134288909250	0.4996600871758710	0.7483671784811059
Ge3	0.1823479758579644	0.1831029689992964	0.1852636984711847
Ge4	0.1881803197043739	0.1818241694680078	0.8166520730870749
Ge5	0.1875716419445217	0.8140218002268406	0.8186492651585777
Ge6	0.8139908073895145	0.1848841782370159	0.1815809272987838
Ge7	0.8141583124382460	0.8173927277162658	0.1861883700001500
Ge8	0.8154255958597660	0.8185517058539961	0.8129810687959278
Ge9	0.3149876123035369	0.6835289861510604	0.6839849460561951
Ge10	0.3156921056578151	0.6841752582112619	0.3129663527508922
Ge11	0.3130870760268394	0.3183724627419956	0.3143330391612645
Ge12	0.6792923851956106	0.3157056053960861	0.6891120545980975
Ge13	0.6860242958237143	0.3165113666476951	0.3187351990640550

Ge14	0.6835426079810558	0.6833967866086368	0.6871361161302483
Ge15	0.1200159681045193	0.9964301312410239	0.6922500979668356
Ge16	0.6893631312305591	0.1173068700381857	0.9985364001966318
Ge17	0.0014196554791672	0.3101970763502753	0.1164756290497371
Ge18	0.9967375156676356	0.6912546563111897	0.1166424592415501
Ge19	0.0025766306521945	0.3062039291024387	0.8804043227789055
Ge20	0.9972570556135059	0.6911578468427441	0.8801350850167543
Ge21	0.8809513485915499	0.0023111495177875	0.3108618683983367
Ge22	0.8843647350658905	0.0005650608803270	0.6876459332252342
Ge23	0.3830429180358014	0.8087191187056354	0.5009376374807729
Ge24	0.3827684437154310	0.1898290556031942	0.4961411077507720
Ge25	0.5022995884108747	0.3822412049034983	0.1914220355957041
Ge26	0.8138336857017964	0.4980348456509657	0.3846015191179155
Ge27	0.1879911340778400	0.5035915563231871	0.3805557505525427
Ge28	0.8074034311404200	0.4960493306712509	0.6208632914166897
Ge29	0.5062543202261495	0.6187101361786095	0.1942824293293634
Ge30	0.6187571784526520	0.8073816373564136	0.5028031570828467
Ba1	0.0035939452945074	0.9991586786198651	0.0023553431534890
Ba2	0.4973396447051572	0.4970584381672066	0.5050096083881842
Ba3	0.2590963508493673	0.5078139588002641	0.0033356864438420
Ba4	0.7391616012675084	0.4844708513824796	0.9878887385878816
Ba5	0.0186237741967006	0.2451758576617905	0.5031927488328862
Ba6	0.9993750991333323	0.7576496425011875	0.4953714834322252
Ba7	0.4976103165180299	0.0006092139270551	0.2440276500664489
Ba8	0.4907514812883325	0.0185309125765720	0.7524621659475192

Supplementary Table 2: Structural details of disorder model of $Ba_8Ga_{16}Ge_{30}$. The atomic positions were obtained by the quasi-random structure approach with a cubic unit cell of lattice parameter 10.74 Å. The Gibbs free energy of this structure is about 25 meV at^{-1} lower than the fully ordered configuration (at 0 K), which will even increase further with increasing temperature due to configurational entropy.

Supplementary Figures

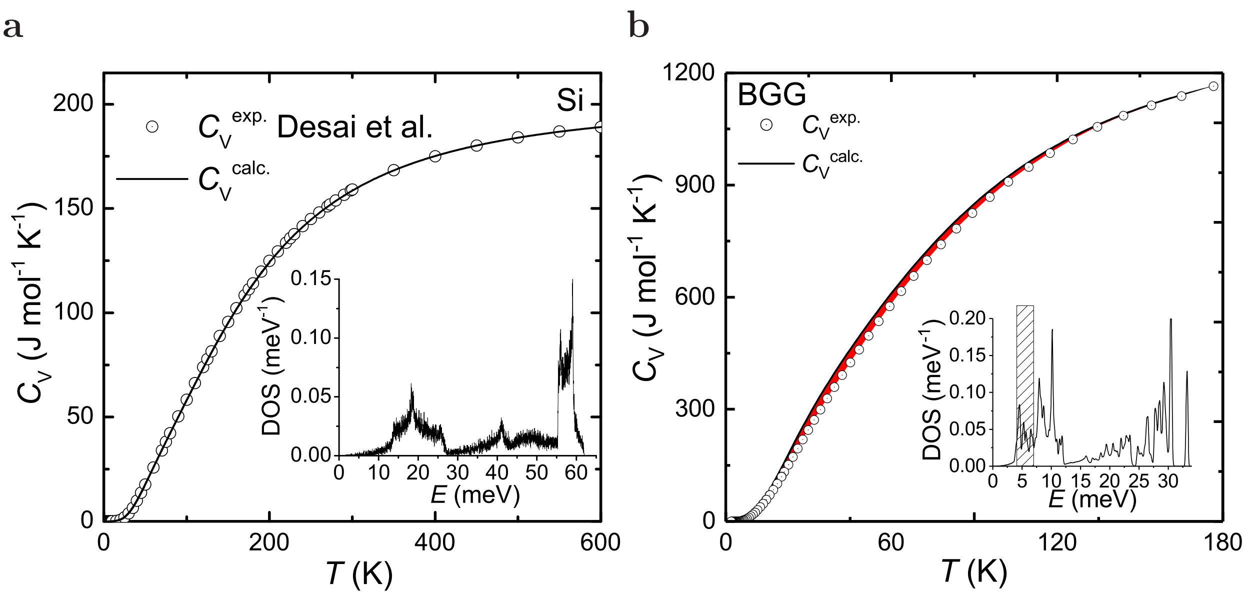

Supplementary Figure 1: Specific heat of Si and $Ba_8Ga_{16}Ge_{30}$. Experimental (symbols) and calculated (lines) specific heat of (**a**) Si and (**b**) $Ba_8Ga_{16}Ge_{30}$ (BGG) versus temperature. Whereas for Si, the calculated specific heat is in good agreement with experimental data[18,19], for $Ba_8Ga_{16}Ge_{30}$ an extra contribution (red area) is observed between 10 and 150 K. As discussed in the main part, this contribution can be attributed to the weakening of the correlation between acoustic and optical phonon modes. The inset in both panels shows the *ab initio* phonon density of states (DOS) as a function of phonon energy, used for calculating the specific heat (see Methods and Supplementary Discussion S6).

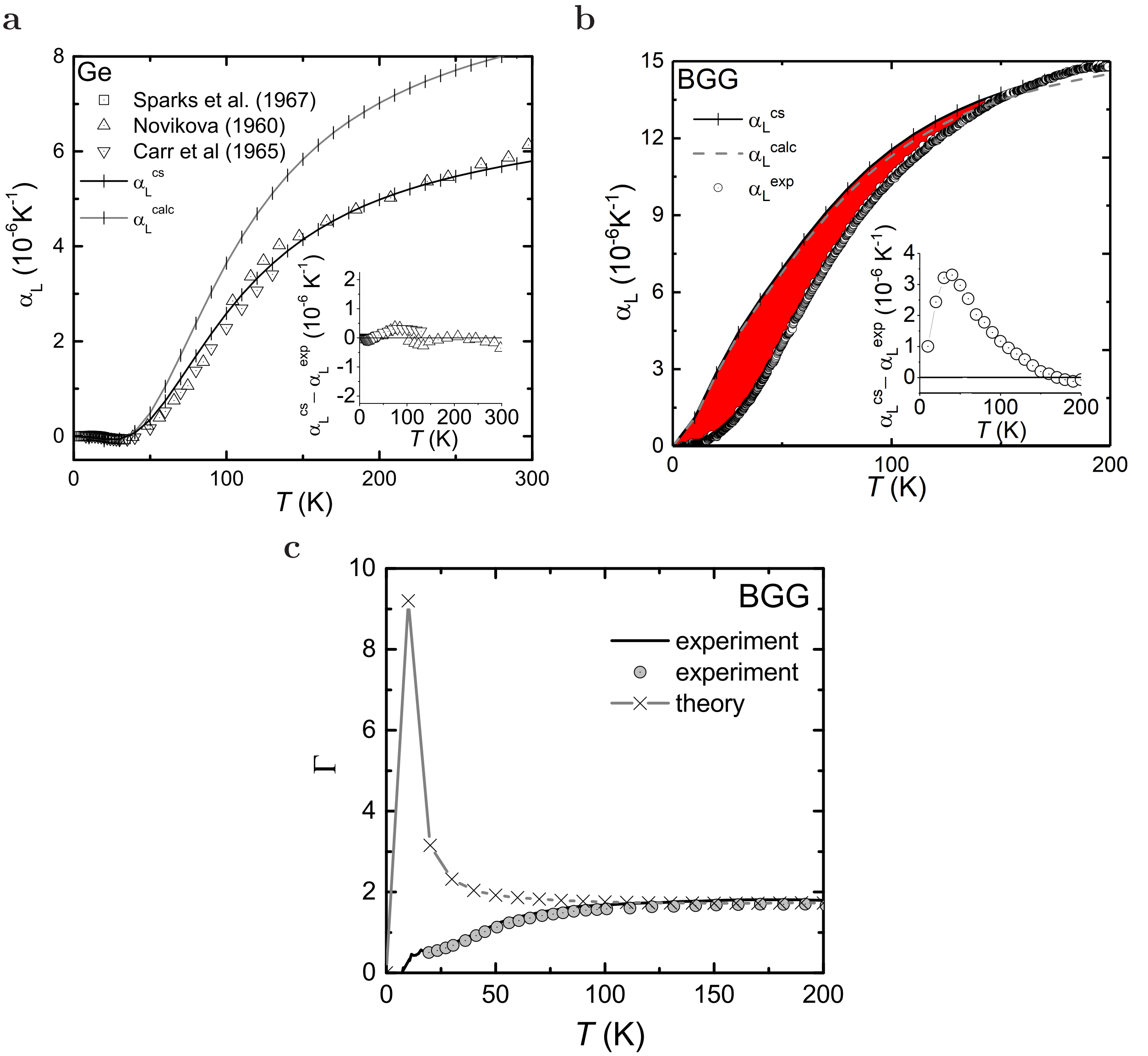

Supplementary Figure 2: Thermal expansion of Ge and $Ba_8Ga_{16}Ge_{30}$, and Grüneisen parameter of $Ba_8Ga_{16}Ge_{30}$. Experimental (α_L^{exp}, symbols), calculated (α_L^{calc}, gray lines), and rescaled (α_L^{cs}, black lines) thermal expansion of (**a**) Ge and (**b**) $Ba_8Ga_{16}Ge_{30}$ (BGG) versus temperature. Whereas for Ge, the calculated (and rescaled) thermal expansion is in good agreement with experimental data[66–68], for $Ba_8Ga_{16}Ge_{30}$ an extra contribution (red area) is observed below 150 K. The difference between theory and experiment is plotted in the two insets. (**c**) Comparison of the experimental Grüneisen parameter $\Gamma = B\alpha_V/C_V$ of $Ba_8Ga_{16}Ge_{30}$, using either a temperature-independent bulk modulus B of 65 GPa (black solid line) or the temperature-dependent one of $Eu_8Ga_{16}Ge_{30}$ (Ref. 69, gray circles), and the theoretical Grüneisen parameter determined from our *ab initio* lattice dynamics calculations (crosses and gray line). C_V is the specific heat at constant volume, α_V is the volumetric thermal expansion coefficient.

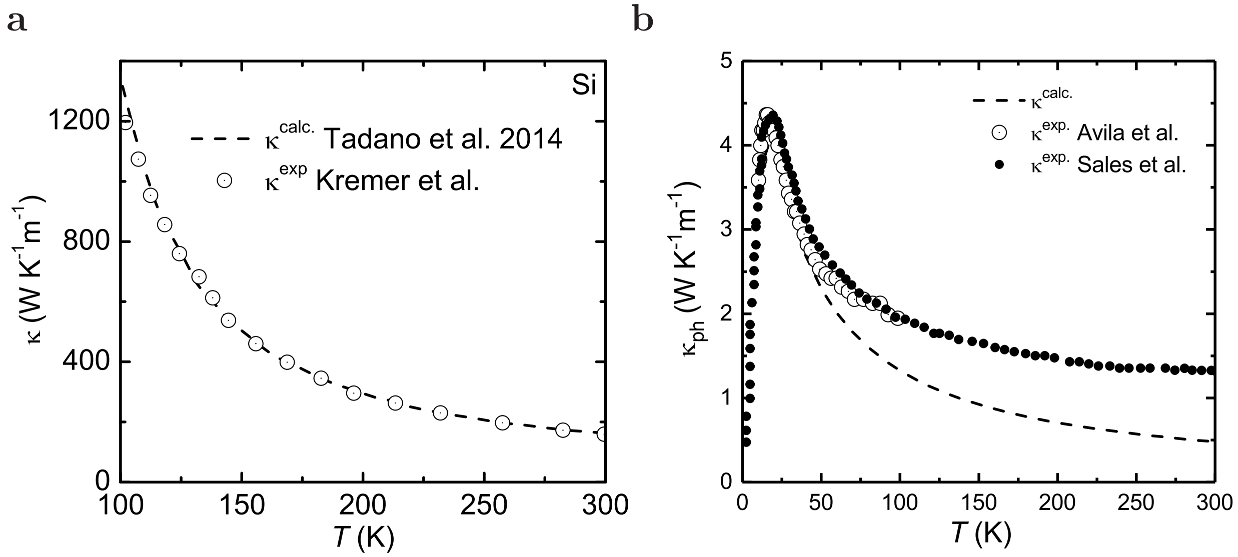

Supplementary Figure 3: Thermal conductivity of Si and $Ba_8Ga_{16}Ge_{30}$. Experimental (symbols) and calculated (dashed lines) thermal conductivity of (**a**) Si and (**b**) $Ba_8Ga_{16}Ge_{30}$ (BGG) versus temperature. While for Si, the calculated thermal conductivity[49] is in good agreement with experimental data[70], for $Ba_8Ga_{16}Ge_{30}$ the calculated thermal conductivity undershoots the experimental data[56,71] significantly above 50 K. As discussed in the main part, the inverse thermal conductivity difference shows approximate $-\ln T$ behaviour, the hallmark of incoherent Kondo scattering in spin Kondo systems.

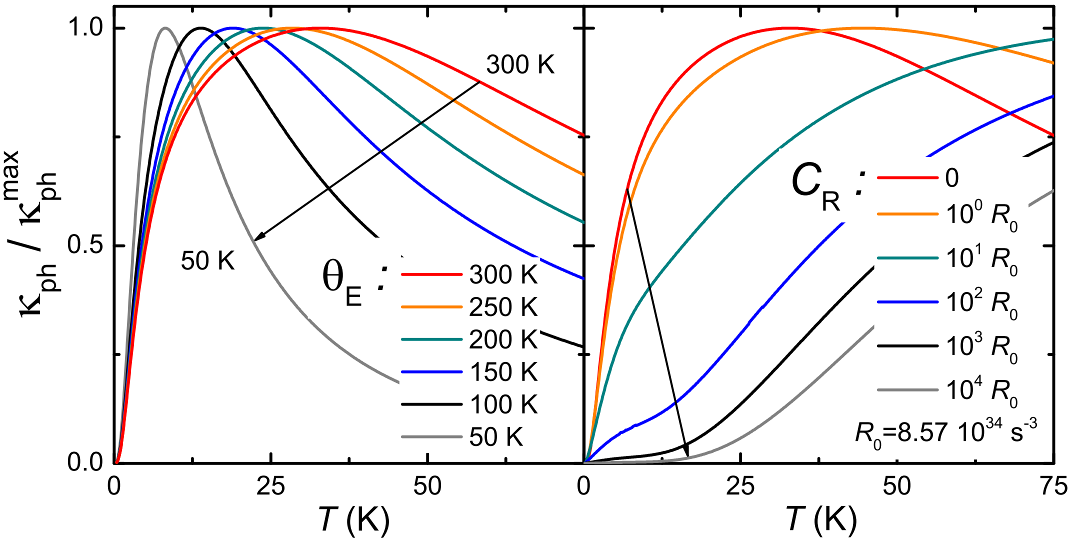

Supplementary Figure 4: Enhanced Umklapp versus resonance scattering. Temperature dependent phonon thermal conductivity, normalized to its maximum, calculated using a modified Callaway model for various Einstein temperatures Θ_E (left, replotted from Fig. 3b left) and for the $\Theta_E = 300\,\mathrm{K}$ curve modified by resonance scattering[72] according to $\tau_R^{-1} = \frac{C_R \omega^2}{\left(\omega_0^2 - \omega^2\right)^2}$, where ω_0 is the circular frequency of the lowest-lying optical mode, with different scattering levels C_R (right). Whereas enhanced Umklapp scattering leads to a sharpening of the phonon thermal conductivity maximum at low temperatures and a suppression of the thermal conductivity in a wide temperature range above the maximum, resonance scattering leads to an additional shoulder and a reduction of the thermal conductivity at low temperatures. Resonance scattering can thus be ruled out as an important scattering channel for the type-I clathrates studied here.

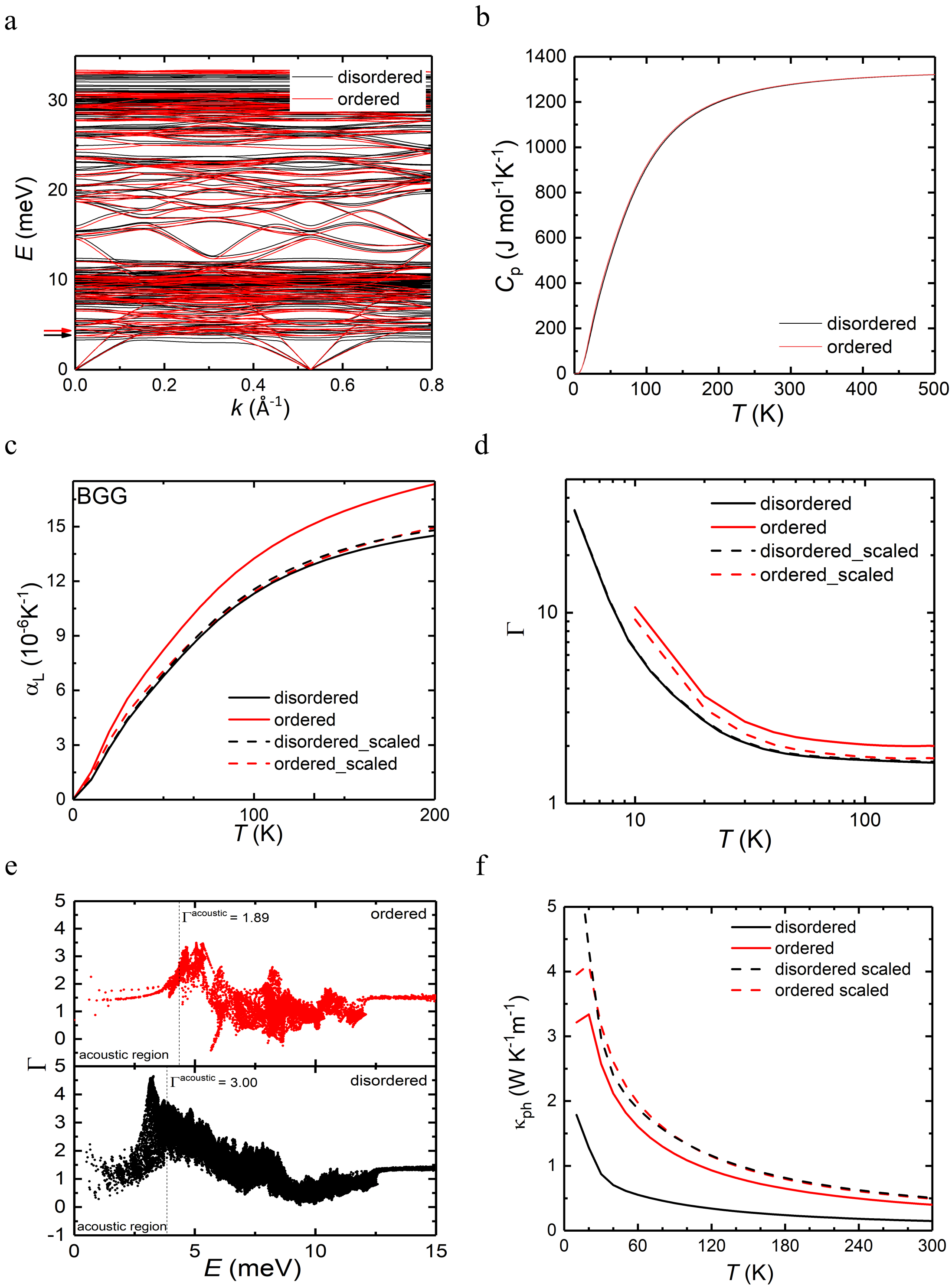

Supplementary Figure 5: Ordered versus disordered Ga configuration in $Ba_8Ga_{16}Ge_{30}$. Comparison of our DFT calculations for (**a**) the phonon dispersion, (**b**) specific heat, (**c**) thermal expansion, (**d**) mode-averaged Grüneisen parameter, (**e**) mode-specific Grüneisen parameter at $T = 0$, and (**f**) thermal conductivity, performed all for a fully ordered model and for a model that takes Ga/Ge site disorder into account (see Sup-

plementary Discussion S9). The arrows in **a** indicate the energy of the lowest-lying optical mode at the border of the Brillouin zone. These energies were used as the upper boundary of the acoustic regime in **e**. The specific heat results agree within 1% at 100 K (see **b**). The thermal expansion curves, scaled to experimental data at 200 K (Supplementary Fig. 2b), virtually fall on top of each other (dashed curves in **c**, 1% deviation at 100 K). The temperature-dependent (mode-averaged) Grüneisen parameter, calculated from these scaled thermal expansion curves, acquires a difference of less than 3% at 100 K (dashed curves in **d**). The mode-specific Grüneisen parameter is similar for both models, except for the larger spread in the low-energy data for the disordered model, which is due to the lower symmetry. The mode-averaged Grüneisen parameter for the acoustic regime (dashed lines indicate its upper boundary, determined as the energy of the lowest-lying optical mode), Γ^{acoustic}, is larger for the disordered model (see **e**), in agreement with the smaller absolute value of the thermal conductivity. The temperature dependence of the thermal conductivity is unaffected by the disorder, as seen by the collapse of the scaled curves (dashed lines in **f**; scaled to match experimental thermal conductivity at low temperatures). These results demonstrate that the temperature dependences of thermodynamic and thermal transport properties of $Ba_8Ga_{16}Ge_{30}$ are robust against disorder.

Supplementary References

[1] Callaway, J. Model for lattice thermal conductivity at low temperatures. *Phys. Rev.* **113**, 1046–1051 (1959).

[2] Euchner, H., Pailhès, S., Nguyen, L. T. K., Assmus, W., Ritter, F., Haghighirad, A., Grin, Y., Paschen, S. & de Boissieu, M. Phononic filter effect of rattling phonons in the thermoelectric clathrate $Ba_8Ge_{40+x}Ni_{6-x}$. *Phys. Rev. B* **86**, 224303 (2012).

[3] Pailhès, S., Euchner, H., Giordano, V. M., Debord, R., Assy, A., Gomès, S., Bosak, A., Machon, D., Paschen, S. & de Boissieu, M. Localization of propagative phonons in a perfectly crystalline solid. *Phys. Rev. Lett.* **113**, 025506 (2014).

[4] Asen-Palmer, M., Bartkowski, K., Gmelin, E., Cardona, M., Zhernov, A. P., Inyushkin, A. V., Taldenkov, A., Ozhogin, V. I., Itoh, K. M. & Haller, E. E. Thermal conductivity of germanium crystals with different isotopic compositions. *Phys. Rev. B* **56**, 9431–9447 (1997).

[5] Klemens, P. *Encyclopedia of Physics*, chap. Thermal Conductivities of Solids at Low Temperatures, 198–281 (ed. S. Flügge, Springer Verlag, 1956).

[6] M. Ikeda. Ph.D. thesis, Mechanism of the thermal conductivity of type-I clathrates, Vienna University of Technology, Austria (2015).

[7] Klemens, P. G. The thermal conductivity of dielectric solids at low temperatures. *Proc. Roy. Soc. (London) A* **208**, 108–133 (1951).

[8] Christensen, S., Schmøkel, M. S., Borup, K. A., Madsen, G. K. H., McIntyre, G. J., Capelli, S. C., Christensen, M. & Iversen, B. B. "Glass-like" thermal conductivity gradually induced in thermoelectric $Sr_8Ga_{16}Ge_{30}$ clathrate by off-centered guest atoms. *J. Appl. Phys.* **119**, 185102 (2016).

[9] Slack, G. A. & Galginaitis, S. Thermal conductivity and phonon scattering by magnetic impurities in CdTe. *Phys. Rev.* **133**, A253–A268 (1964).

[10] Pippard, A. B. Ultrasonic attenuation in metals. *Phil. Mag.* **46**, 1104–1114 (1955).

[11] Cahill, D. G., Watson, S. K. & Pohl, R. O. Lower limit to the thermal conductivity of disordered crystals. *Phys. Rev. B* **46**, 6131–6140 (1992).

[12] Bryant, C. A. & Keesom, P. H. Low-temperature specific heat of germanium. *Phys. Rev.* **124**,

698–700 (1961).

[13] Bentien, A., Christensen, M., Bryan, J. D., Sanchez, A., Paschen, S., Steglich, F., Stucky, G. D. & Iversen, B. B. Thermal conductivity of thermoelectric clathrates. *Phys. Rev. B* **69**, 045107 (2004).

[14] Yan, X., Grytsiv, A., Giester, G., Bauer, E., Rogl, P. & Paschen, S. Single-crystal investigations on quaternary clathrates $Ba_8Cu_5Si_xGe_{41-x}$ (x = 6, 18, 41). *J. Electron. Mater.* **40**, 589–596 (2011).

[15] Sui, F., He, H., Bobev, S., Zhao, J., Osterloh, F. E. & Kauzlarich, S. K. Synthesis, structure, thermoelectric properties, and band gaps of alkali metal containing type I clathrates: $A_8Ga_8Si_{38}$ (A = K, Rb, Cs) and $K_8Al_8Si_{38}$. *Chem. Mater.* **27**, 2812–2820 (2015).

[16] Rogl, G., Grytsiv, A., Rogl, P., Royanian, E., Bauer, E., Horky, J., Setman, D., Schafler, E. & Zehetbauer, M. Dependence of thermoelectric behaviour on severe plastic deformation parameters: A case study on p-type skutterudite $DD_{0.60}Fe_3CoSb_{12}$. *Acta Mater.* **61**, 6778–6789 (2013).

[17] Rogl, G., Grytsiv, A., Bursik, J., Horky, J., Anbalagan, R., Bauer, E., Mallik, R. C., Rogl, P. & Zehetbauer, M. Changes in microstructure and physical properties of skutterudites after severe plastic deformation. *Phys. Chem. Chem. Phys.* **17**, 3715–3722 (2015).

[18] Desai, P. D. Thermodynamic properties of iron and silicon. *J. Phys. Chem. Ref. Data* **15**, 967–983 (1986).

[19] Okada, Y. & Tokumaru, Y. Precise determination of lattice parameter and thermal expansion coefficient of silicon between 300 and 1500 K. *J. Appl. Phys.* **56**, 314–320 (1984).

[20] Hall, J. J. Electronic effects in the elastic constants of n-type silicon. *Phys. Rev.* **161**, 756–761 (1967).

[21] Christensen, M., Lock, N., Overgaard, J. & Iversen, B. B. Crystal structures of thermoelectric n- and p-type $Ba_8Ga_{16}Ge_{30}$ studied by single crystal, multitemperature, neutron diffraction, conventional X-ray diffraction and resonant synchrotron X-ray diffraction. *J. Am. Chem. Soc.* **128**, 15657–15665 (2006).

[22] Okamoto, N., Nakano, T., Tanaka, K. & Inui, H. Mechanical and thermal properties of single crystals of the type-I clathrate compounds $Ba_8Ga_{16}Ge_{30}$ and $Sr_8Ga_{16}Ge_{30}$. *J. Appl. Phys.* **104**, 013529 (2008).

[23] Nguyen, L. T. K., Aydermir, U., Baitinger, M., Bauer, E., Borrmann, H., Burkhardt, U.,

Custers, J., Haghighirad, A., Höfler, R., Luther, K. D., Ritter, F., Assmus, W., Grin, Y. & Paschen, S. Atomic ordering and thermoelectric properties of the n-type clathrate $Ba_8Ni_{3.5}Ge_{42.1}\square_{0.4}$. *Dalton Trans.* **39**, 1071–1077 (2010).

[24] Zhang, H., Borrmann, H., Oeschler, N., Candolfi, C., Schnelle, W., Schmidt, M., Burkhardt, U., Baitinger, M., Zhao, J.-T. & Grin, Y. Atomic interactions in the p-type clathrate I $Ba_8Au_{5.3}Ge_{40.7}$. *Inorg. Chem.* **50**, 1250–1257 (2011).

[25] Zeiringer, I., Chen, M., Bednar, I., Royanian, E., Bauer, E., Podloucky, R., Grytsiv, A., Rogl, P. & Effenberger, H. Phase equilibria, crystal chemistry, and physical properties of Ag-Ba-Si clathrates. *Acta Mater.* **59**, 2368–2384 (2011).

[26] Schäfer, H. On the problem of polar intermetallic compounds: The stimulation of E. Zintl's work for the modern chemistry of intermetallics. *Ann. Rev. Mater. Sci.* **15**, 1–41 (1985).

[27] Kondo, J. Resistance minimum in dilute magnetic alloys. *Progr. Theor. Phys.* **32**, 37–49 (1964).

[28] Hewson, A. C. *The Kondo Problem to Heavy Fermions* (Cambridge University Press, Cambridge, 1997).

[29] Coleman, P. *Introduction to Many-Body Physics* (Cambridge University Press, Cambridge, 2015).

[30] Coqblin, B. & Schrieffer, J. R. Exchange interaction in alloys with cerium impurities. *Phys. Rev.* **185**, 847–853 (1969).

[31] Nozières, P. & Blandin, A. Kondo effect in real metals. *J. Phys.* **41**, 193–211 (1980).

[32] Cox, D. L. Quadrupolar Kondo effect in uranium heavy-electron materials? *Phys. Rev. Lett.* **59**, 1240–1243 (1987).

[33] Wilhelm, U., Schmid, J., Weis, J. & Klitzing, K. Experimental evidence for spinless Kondo effect in two electrostatically coupled quantum dot systems. *Physica E* **14**, 385–390 (2002).

[34] Katayama, S., Maekawa, S. & Fukuyama, H. Kondo-like effect of atomic motion on resistivity in $Pb_{1-x}Ge_xTe$. *J. Phys. Soc. Jpn.* **56**, 697–705 (1987).

[35] Cox, D. L. & Zawadowski, A. Exotic Kondo effects in metals: Magnetic ions in a crystalline electric field and tunnelling centres. *Adv. Phys.* **47**, 599–942 (1998).

[36] Hotta, T. Quasi-Kondo phenomenon due to the dynamical Jahn-Teller effect. *Phys. Rev. Lett.* **96**, 197201 (2006).

[37] Hotta, T. Enhanced Kondo effect in an electron system dynamically coupled with local optical phonons. *J. Phys. Soc. Jpn.* **76**, 084702 (2007).

[38] Withoff, D. & Fradkin, E. Phase transitions in gapless Fermi systems with magnetic impurities. *Phys. Rev. Lett.* **64**, 1835–1838 (1990).

[39] Yanagisawa, T. Kondo effect in Dirac systems. *J. Phys. Soc. Jpn.* **84**, 074705 (2015).

[40] Chen, J.-H., Li, L., Cullen, W. G., Williams, E. D. & Fuhrer, M. S. Tunable Kondo effect in graphene with defects. *Nat. Phys.* **7**, 535–538 (2011).

[41] Duan, L.-M. Controlling ultracold atoms in multi-band optical lattices for simulation of Kondo physics. *EPL* **67**, 721–727 (2004).

[42] Flottat, T., Hébert, F., Rousseau, V. G., Scalettar, R. T. & Batrouni, G. G. Bosonic Kondo-Hubbard model. *Phys. Rev. B* **92**, 035101 (2015).

[43] Read, N. & Newns, D. M. On the solution of the Coqblin-Schrieffer Hamiltonian by the large-N expansion technique. *J. Phys. C* **16**, 3273–3295 (1983).

[44] Coleman, P. Mixed valence as an almost broken symmetry. *Phys. Rev. B* **35**, 5072–5116 (1987).

[45] Arovas, D. P. & Auerbach, A. Functional integral theories of low-dimensional quantum Heisenberg models. *Phys. Rev. B* **38**, 316–332 (1988).

[46] Tadano, T., Gohda, Y. & Tsuneyuki, S. Impact of rattlers on thermal conductivity of a thermoelectric clathrate: A first-principles study. *Phys. Rev. Lett.* **114**, 095501 (2015).

[47] He, Y. & Galli, G. Nanostructured clathrate phonon glasses: Beyond the rattling concept. *Nano Lett.* **14**, 2920–2925 (2014).

[48] Zunger, A., Wei, S.-H., Ferreira, L. G. & Bernard, J. E. Special quasirandom structures. *Phys. Rev. Lett.* **65**, 353–356 (1990).

[49] Tadano, T., Gohda, Y. & Tsuneyuki, S. Anharmonic force constants extracted from first-principles molecular dynamics: applications to heat transfer simulations. *J. Phys.: Condens. Matter* **26**, 225402 (2014).

[50] Chen, C., Zhang, Z. & Chen, J. Revisit to the impacts of rattlers on thermal conductivity of clathrates. *Front. Energy Res.* **6**, 34 (2018).

[51] Lindroth, D. O., Brorsson, J., Fransson, E., Palmqvist, F. E. A., Erhart, P., Thermal conductivity in intermetallic clathrates: A first principles perspective; arXiv:1807.01502.

[52] Christensen, M., Abrahamsen, A., Christensen, N., Juranyi, F., Andersen, N., Lefmann, K., Andreasson, J., Bahl, C. & Iversen, B. Avoided crossing of rattler modes in thermoelectric materials. *Nat. Mater.* **7**, 811–815 (2008).

[53] Lory, P.-F., Pailhès, S., Giordano, V. M., Euchner, H., Nguyen, H. D., Ramlau, R., Borrmann,

H., Schmidt, M., Baitinger, M., Ikeda, M., Tomeš, P., Mihalkovič, M., Allio, C., Johnson, M. R., Schober, H., Sidis, Y., Bourdarot, F., Regnault, L. P., Ollivier, J., Paschen, S., Grin, Y. & de Boissieu, M. Direct measurement of individual phonon lifetimes in the clathrate compound $Ba_{7.81}Ge_{40.67}Au_{5.33}$. *Nat. Commun.* **8**, 491 (2017).

[54] Tomeš, P., Ikeda, M., Eguchi, G., Baitinger, M., Nguyen, H. D., Mihalcovič, M., Popčević, P., Allio, C., Krellner, C., Lory, P.-F., Pailhès, S., Giordano, V. M., Euchner, H., Smontara, A., Barišić, N., de Boissieu, M., Grin, Y., and Paschen, S., Anisotropic transport in a cubic type-I clathrate, unpublished.

[55] Prokofiev, A., Sidorenko, A., Hradil, K., Ikeda, M., Svagera, R., Waas, M., Winkler, H., Neumaier, K. & Paschen, S. Thermopower enhancement by encapsulating cerium in clathrate cages. *Nat. Mater.* **12**, 1096–1101 (2013).

[56] Sales, B. C., Chakoumakos, B. C., Jin, R., Thompson, J. R. & Mandrus, D. Structural, magnetic, thermal, and transport properties of $X_8Ga_{16}Ge_{30}$ (X = Eu, Sr, Ba) single crystals. *Phys. Rev. B* **63**, 245113 (2001).

[57] Qiu, L., Swainson, I. P., Nolas, G. S. & White, M. A. Structure, thermal, and transport properties of the clathrates $Sr_8Zn_8Ge_{38}$, $Sr_8Ga_{16}Ge_{30}$, and $Ba_8Ga_{16}Si_{30}$. *Phys. Rev. B* **70**, 035208 (2004).

[58] Paschen, S., Carrillo-Cabrera, W., Bentien, A., Tran, V. H., Baenitz, M., Grin, Y. & Steglich, F. Structural, transport, magnetic, and thermal properties of $Eu_8Ga_{16}Ge_{30}$. *Phys. Rev. B* **64**, 214404–214414 (2001).

[59] Avila, M. A., Suekuni, K., Umeo, K., Fukuoka, H., Yamanaka, S. & Takabatake, T. $Ba_8Ga_{16}Sn_{30}$ with type-I clathrate structure: Drastic suppression of heat conduction. *Appl. Phys. Lett.* **92**, 041901 (2008).

[60] Ishii, I., Suetomi, Y., Fujita, T. K., Suekuni, K., Tanaka, T., Takabatake, T. & Suzuki, T. Lattice instability and elastic dispersion due to the rattling motion in the type-I clathrate $Ba_8Ga_{16}Sn_{30}$. *Phys. Rev. B* **85**, 085101 (2012).

[61] Saiga, Y., Suekuni, K., Du, B. & Takabatake, T. Thermoelectric properties and structural instability of type-I clathrate $Ba_8Ga_{16}Sn_{30}$ at high temperatures. *Solid State Commun.* **152**, 1902–1905 (2012).

[62] Gabitto, J. F. & Tsouris, C. Physical properties of gas hydrates: A review. *J. Thermodyn.* **2010**, 271291 (2010).

[63] Handa, Y. P. & Cook, J. G. Thermal conductivity of xenon hydrate. *J. Phys. Chem.* **91**, 6327–6328 (1987).

[64] Tse, J. S., Shpakov, V. P., Belosludov, V. R., Trouw, F., Handa, Y. P. & Press, W. Coupling of localized guest vibrations with the lattice modes in clathrate hydrates. *Europhys. Lett.* **54**, 354–360 (2001).

[65] Chetty, R., Kumar D. S., P., Rogl, G., Rogl, P., Bauer, E., Michor, H., Suwas, S., Puchegger, S., Giester, G. & Malik, R. C. Thermoelectric properties of a Mn substituted synthetic tetrahedrite. *Phys. Chem. Chem. Phys.* **17**, 1716–1727 (2015).

[66] Carr, R. H., McCammon, R. D. & White, G. K. Thermal expansion of germanium and silicon at low temperatures. *Philos. Mag.* **12**, 157–163 (1965).

[67] Sparks, P. W. & Swenson, C. A. Thermal expansions from 2 to 40 K of Ge, Si, and four III-V compounds. *Phys. Rev.* **163**, 779–790 (1967).

[68] Novikova, S. I. Thermal expansion of Ge at low temperatures (Russian). *Fiz. Tverd. Tela* **2**, 43–44 (1960).

[69] Zerec, I., Keppens, V., McGuire, M., Mandrus, D., Sales, B. & Thalmeier, P. Four-well tunneling states and elastic response of clathrates. *Phys. Rev. Lett.* **92**, 185502 (2004).

[70] Kremer, R. K., Graf, K., Cardona, M., Devyatykh, G. G., Gusev, A. V., Gibin, A. M., Inyushkin, A. V., Taldenkov, A. N. & Pohl, H. Thermal conductivity of isotopically enriched ^{28}Si: revisited. *Solid State Commun.* **131**, 499–503 (2004).

[71] Avila, M. A., Suekuni, K., Umeo, K., Fukuoka, H., Yamanaka, S. & Takabatake, T. Glasslike versus crystalline thermal conductivity in carrier-tuned $Ba_8Ga_{16}Ge_{30}$. *Phys. Rev. B* **74**, 125109 (2006).

[72] Pohl, R. O. Thermal conductivity and phonon resonance scattering. *Phys. Rev. Lett.* **8**, 481–483 (1962).
